# Fast and robust feature-based stitching algorithm for microscopic images

**DOI:** 10.1038/s41598-024-61970-y

**Published:** 2024-06-10

**Authors:** Fatemeh Sadat Mohammadi, Hasti Shabani, Mojtaba Zarei

**Affiliations:** 1 Institute of Medical Science and Technology, Shahid Beheshti University, Tehran, Iran; 2 Department of Clinical Research, University of Southern Denmark, Odense, Denmark

**Keywords:** Microscopic image, Whole-slide image, Image stitching, Pairwise registration, Global alignment, Biomedical engineering, Image processing, Software

## Abstract

The limited field of view of high-resolution microscopic images hinders the study of biological samples in a single shot. Stitching of microscope images (tiles) captured by the whole-slide imaging (WSI) technique solves this problem. However, stitching is challenging due to the repetitive textures of tissues, the non-informative background part of the slide, and the large number of tiles that impact performance and computational time. To address these challenges, we proposed the Fast and Robust Microscopic Image Stitching (FRMIS) algorithm, which relies on pairwise and global alignment. The speeded up robust features (SURF) were extracted and matched within a small part of the overlapping region to compute the transformation and align two neighboring tiles. In cases where the transformation could not be computed due to an insufficient number of matched features, features were extracted from the entire overlapping region. This enhances the efficiency of the algorithm since most of the computational load is related to pairwise registration and reduces misalignment that may occur by matching duplicated features in tiles with repetitive textures. Then, global alignment was achieved by constructing a weighted graph where the weight of each edge is determined by the normalized inverse of the number of matched features between two tiles. FRMIS has been evaluated on experimental and synthetic datasets from different modalities with different numbers of tiles and overlaps, demonstrating faster stitching time compared to existing algorithms such as the Microscopy Image Stitching Tool (MIST) toolbox. FRMIS outperforms MIST by 481% for bright-field, 259% for phase-contrast, and 282% for fluorescence modalities, while also being robust to uneven illumination.

## Introduction

Microscopic examination of the tissue is often considered the gold standard in medical diagnosis. This process involves cutting the tissue into thin slices, placing it under a microscope, and observing/analyzing the morphological characteristics. Microscopes have a limited field of view (FOV) due to the high magnification. To solve this issue, some structural improvements have been developed in optical microscopes^1–4^. However, pathologists still need to look at hundreds of images to examine the entire tissue on the glass slide. The whole-slide imaging (WSI) scanner automatically obtains a high optical resolution image at each FOV and continues until it covers the entire tissue on the glass slide. These images (tiles) that have a predefined overlap with each other need to be stitched next to each other to form an image of the entire tissue. For this purpose, different image processing techniques such as illumination correction, stitching, and blending are required to achieve a reliable image from the biological tissues captured by a WSI scanner. The main part of the stitching algorithm is pairwise registration, which estimates the transformation parameters between two adjacent tiles according to the overlapping region. However, there are multiple challenges in stitching microscopic images that may not be the case in computer vision applications: 1) the presence of a repetitive structure in tiles that causes incorrect matching of tiles, 2) the presence of empty regions (lack of texture) in the background tiles, where there is no or very little information about the overlapping region that causes the solution to be ill-posed, and 3) the high number of tiles that propagates error in the final image and increases the stitching time. Therefore, pairwise registration is not sufficient, and designing and implementing a stitching algorithm to overcome the aforementioned challenges and provide a whole-slide image that is consistent and globally registered in the shortest time is crucial in digital microscopy.

Conventionally, the microscopic images are stitched semi-automatically i.e., the user is requested to do the initial matching manually before the pairwise registration^5,6^. In fully automatic approaches, some techniques compute pairwise registration for all possible pairs of tiles to remove the need for manual interference by the user. However, these approaches do not apply to a large number of tiles because of the high computational load^7,8^. In the most recent studies of the automatic stitching algorithm, pairwise registration is done only between the tiles that are adjacent to each other based on the information of the scanning pattern and movement of the microscope motors, in which the computational load is significantly reduced^9–13^. Furthermore, the stitching algorithms can be categorized into three groups depending on the processing involved in the pairwise registration: region-based, feature-based, and a hybrid approach that combines both techniques. In region-based methods, transformation parameters are obtained by moving the images relative to each other and computing the similarity of the images according to the correlation criterion^9–12,14^. In feature-based methods, salient feature points are extracted from images and matched to compute the transformation parameters^15–17^. This method is more accurate and robust to the brightness variance of tiles, but computationally expensive. In combined methods, the feature points are detected, and the computation of the correlation is restricted in local regions around the detected feature points so that the registration computation is sped up^13,18^.

Recent stitching algorithms have been designed to address the computational time and global alignment of microscopic images from different modalities. Chalfoun et al. presented the Microscopy Image Stitching Tool (MIST) toolbox for stitching microscopic images from bright-field, phase-contrast, fluorescence, and Focused Ion Beam-Scanning Electron Microscope (FIB-SEM) modalities^9^. This algorithm uses the phase correlation and normalized cross-correlation (NCC) in pairwise registration. The transformations whose NCC value is less than the threshold or are unacceptable according to the mechanical stage model parameters are filtered and replaced with the median value of the correct transformations. Then, they are optimized by the Constrained Hill-Climbing algorithm. The weighted maximum spanning tree algorithm is used for global alignment. This toolbox utilizes multicore hybrid CPU/GPU implementation, which significantly speeds up the stitching process. This algorithm is faster and provides less error compared to Terastitcher^14^, iStitch^8^, and FijiIs^19^ tools^9^. Seo et al. presented a robust algorithm despite the noise and a small amount of overlap between adjacent tiles of the fluorescence microscopic images^10^. In this study, noise removal and thresholding are performed to detect the presence of cells in tiles and improve the pairwise registration that is based on the NCC. The global alignment is done by grouping tiles with the highest NCC value. Therefore, the tiles with no cells in overlapping regions have the least impact on creating the final image. This algorithm requires less initial information and outperforms MIST^9^ and FijiIs^19^ toolboxes in terms of error, particularly for images with narrow overlap, despite longer stitching time^10^.

Perrot et al. presented a method using template matching in pairwise registration but only applied it to bright-field images. They used template matching only in the overlapping regions of tiles, leading to a reduction in computational load and an enhancement in the algorithm’s speed. For the global alignment, a minimization problem using a weighted graph was solved, in which three approaches were provided for the edges' weight: unit weights, constant weights, and dynamic weights. They concluded that the quality of the stitched image using the dynamic weight was better than the constant weight and the MIST toolbox. Moreover, the dynamic weight method stitched the images faster than the MIST toolbox^12^. In another study, Pellikka et al. presented a method for stitching high-volume images that is robust to repetitive patterns and regions without features in the tiles. To compute possible pairwise correspondences between tiles, feature points were obtained using the Harris corner detector. Then, the local maxima of the Pearson correlation coefficient were computed only in the neighborhood regions of feature points. Global alignment was applied to solve a nonlinear minimization problem with a linear equality constraint. This research did not report the stitching time^13^. Muhlich et al. presented the Alignment by Simultaneous Harmonization of Layer/Adjacency Registration (ASHLAR) Python toolbox for stitching and registering multi-cycle fluorescence images of tissue and cells^11^. The pairwise registration is done using phase correlation and NCC with an accuracy of 0.1 sub-pixels. The low-quality transformations with a weight greater than the threshold or outside the translation limit specified by the user are discarded. The global alignment is done using the minimum spanning tree. Then, the position of these tiles is estimated by multiple linear regression based on the known tile positions. The error rate of the ASHLAR algorithm is similar to the MIST method, but MIST runs faster with a single CPU without using a GPU. ASHLAR stitching performance is also evaluated on bright-field images and reported to have less error compared to three commercial scanners^11^.

We proposed the Fast and Robust Microscopic Image Stitching (FRMIS) algorithm, which is fundamentally based on the fact that microscopic images provide rich feature points due to the texture of the samples and cells: extracting dominant feature points from a small part of the overlapping region reduces the computational time, using invariant local image features makes the stitching algorithm robust, and considering the number of feature points provides less misalignment globally in the generated whole-slide image.

## Materials and methods

### Materials

**Figure 1:**
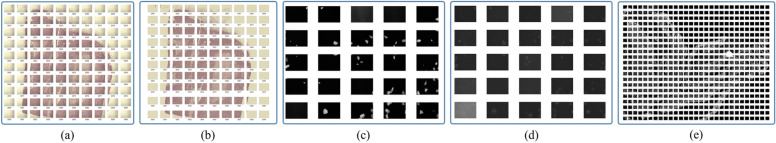
Examples of image sets: (**a**) the original 49–01 image set from the Tak dataset with shading, (**b**) the corrected 49–01 image set from the Tak dataset, (**c**) the image set from the stem cell colony dataset with fluorescence modality, (**d**) the image set from the stem cell colony dataset with phase-contrast modality, and (e) the image set from human colon dataset.

To evaluate the proposed stitching algorithm, multiple experimental microscopic datasets from different modalities with varying numbers of tiles and overlaps were used: 1) the collection of bright-field images was prepared by Tak et al.^20^, which includes ten samples of different cells with different densities. This collection contains two sets of tiles for each sample: the set of tiles that were the direct output of the light microscope with shading and fixed-pattern noise, and the set of tiles that were corrected using uneven-illumination correction methods, which is one of the most important steps in creating whole-slide images, though not the purpose of this study. Both sets of tiles were utilized in this study to evaluate the proposed FRMIS algorithm. 2) The collection of stem cell colony dataset^21^ includes images from the phase-contrast and fluorescence modalities. 3) The collection of images of normal human colon samples^22^ has been obtained using the fluorescence modality, with a small overlap region between tiles. Figure 1 displays examples of the image set from the experimental datasets.

Since the experimental image collections do not have a ground truth to evaluate the final stitched result precisely, we generated two synthetic datasets by dividing the bright-field breast histology whole-slide images of the ICIAR dataset^23^ and the USAF optical microscope resolution test slides into a 10 × 10 grid with a 30% overlap. We chose USAF images, which are binary images including sharp corners and empty regions. These images are usually used to test the resolution of microscopes. Table 1 summarizes the characteristics of the image datasets that were used in this study.

**Table 1:** Summary of the microscopic image datasets used in this study.

Dataset		Tiles (grid)	Magnification	Size of tile	Overlap	Modality	Samples
*Experimental*	Tak^20^	100 (10 × 10)	40 ×	2304 × 1719	25%	Bright-field	10
	Stem cell colony^21^	552 (23 × *24),* *100 (10* × *10),* 25 (5 × 5)	10 ×	1392 × 1040	*10% or* 19%	Phase-contrast and Fluorescence	6
	Human colon^22^	609 (29 × 21)	20 ×	1280 × 1080	2% to 3%	Fluorescence	1
Synthetic	ICIAR^23^	100 (10 × 10)	5 ×	Different size	30%	Bright-field	6
	USAF	100 (10 × 10)	-	Different size	30%	-	6

## Methods

**Figure 2:**
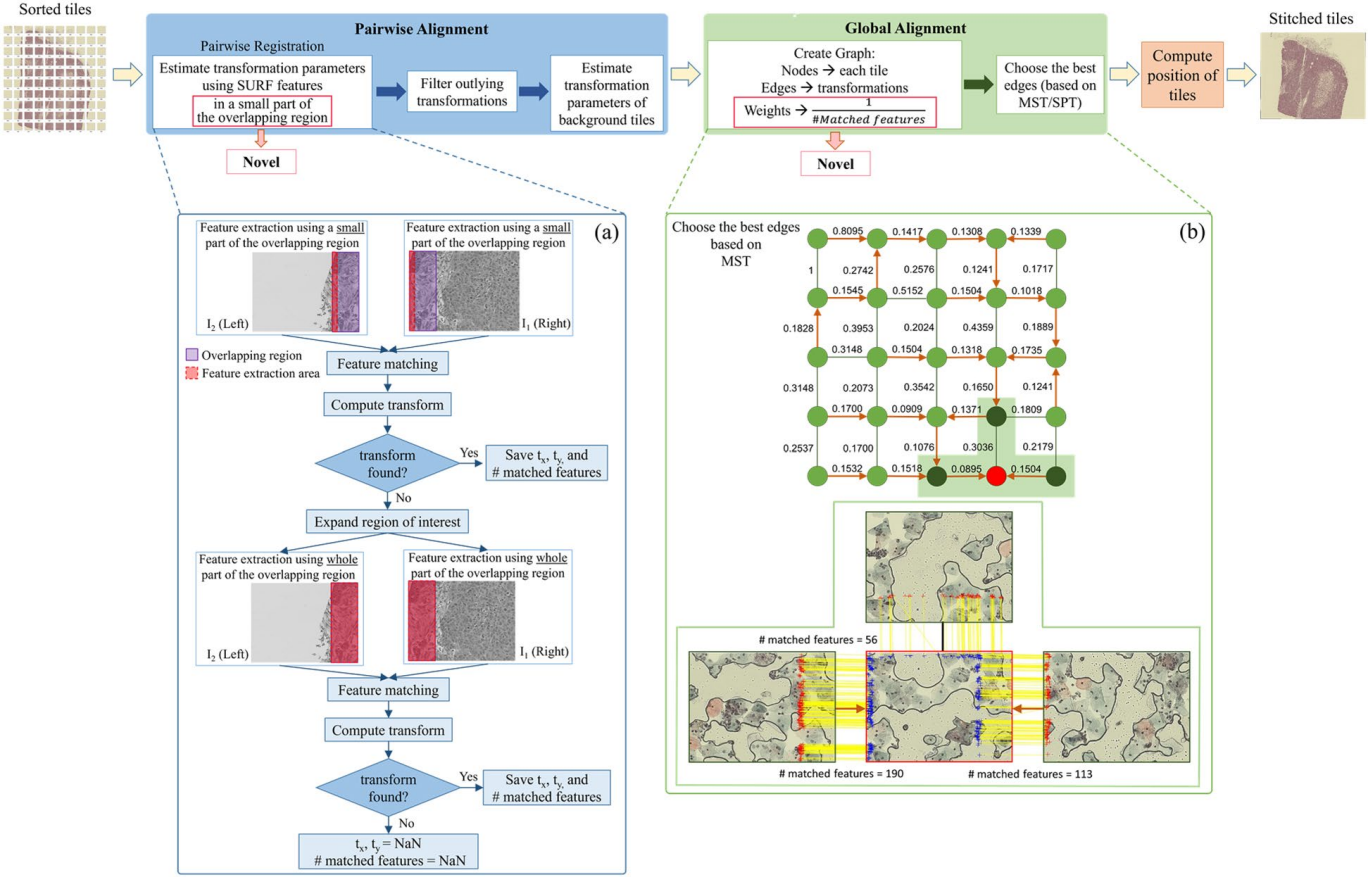
Block diagram of the FRMIS algorithm, (**a**) the block diagram of the pairwise registration algorithm in the west direction, and (**b**) a sample graph with the computed weight of each edge in the global alignment step.

The FRMIS algorithm involves two main steps as shown in Fig. 2: pairwise and global alignment. The scan pattern in the WSI technique reveals the connection order of tiles and is used to sort them next to each other in a predefined pattern. Therefore, the purpose of the pairwise alignment is to compute the transformation parameters of each tile (textured tile and/or background tile) only with adjacent north and west tiles. In the pairwise registration, the speeded up robust feature (SURF) key points were extracted and matched only in a small part of the overlapping region. If the transformation could not be computed due to an insufficient number of matched features, features were extracted from the entire overlapping region. Then, a weighted connected graph using the minimum spanning tree (MST) or the shortest path tree (SPT) was created in the global alignment step. In this step, the weight of the edges in the graph is designed to reduce the overall error, taking into account the number of matched features. Finally, the position of each tile was determined, and tiles were placed within the frame to generate the final stitched image. Each step is shown in Fig. 2 and explained in detail as follows.

### Pairwise alignment

**Figure 3:**
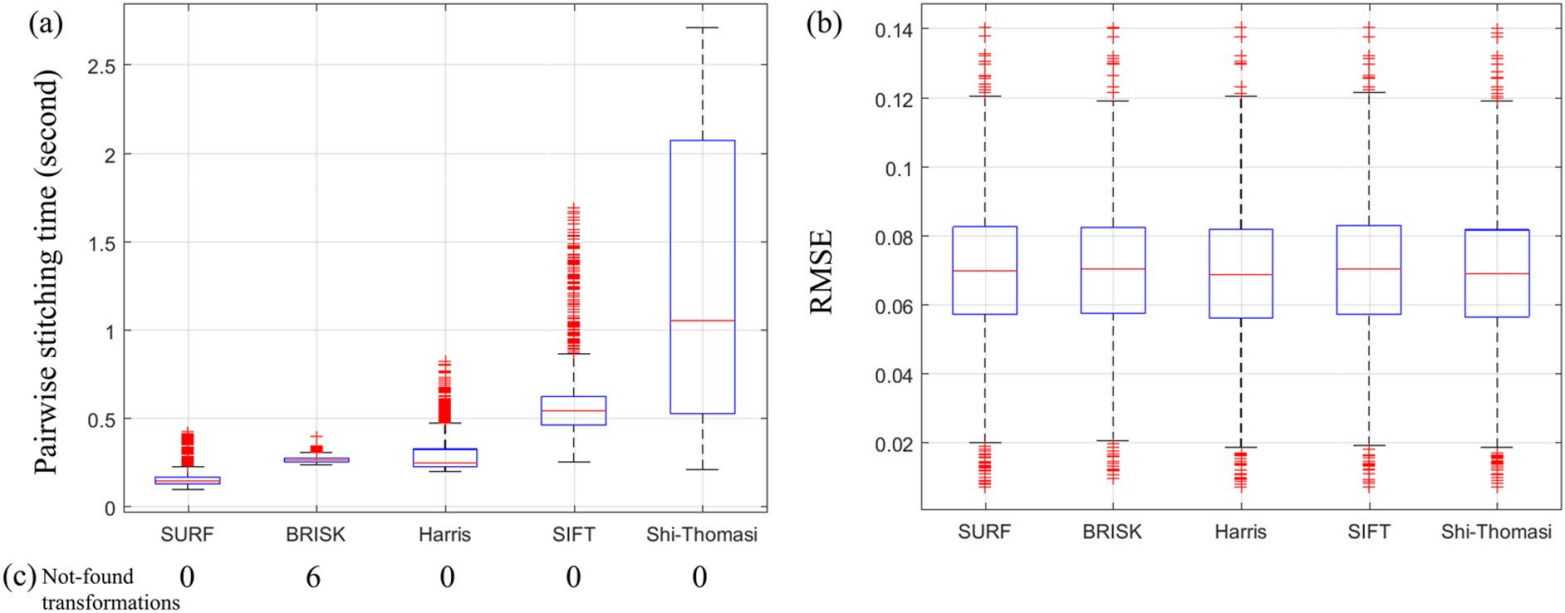
Comparison of the performance of SURF, BRISK, Harris, SIFT, and Shi-Thomasi features in pairwise registration: (**a**) pairwise stitching time, (**b**) RMSE between the intensity of pixels in the overlapping region of adjacent tiles, and (**c**) the number of fails to compute the transformations of 1603 non-background tile pairs from Tak dataset.

The main part of the FRMIS algorithm is to estimate the transformation parameters between two adjacent tiles using the feature points extracted from the overlapping region. Unique and stable key feature points directly impact the accurate computation of the transformation parameters between the adjacent tiles and the quality of the final stitched image. Various feature detection methods have been used in literature, and SIFT^24^, SURF^25^, Harris^26^, BRISK^27^, and Shi-Thomasi^28^ are the most popular. We investigated these feature points in terms of accuracy and processing time in pairwise registration to select the best one. For this purpose, we used the Tak dataset and chose tile pairs in which the overlapping region of tiles contains texture, not background (1603 out of 1800 tile pairs were selected). Figure 3 illustrates the root-mean-square error (RMSE) of the pixel intensity in the overlapping region of two adjacent tiles, the required time to compute transformation parameters between them, and the number of failed attempts to compute the transformation referred to as “not-found transformations”. Comparing these feature points shows that the SURF^23^ method runs faster than other features (Fig. 3a), while its accuracy is comparable with other feature points (Fig. 3b). We also found that SURF, SIFT, and Shi-Thomasi successfully found transformations for all pair of tiles, while BRISK failed to compute the transformation for six pair of non-background tiles (Fig. 3c). In addition, the authors conducted a comprehensive study on pairwise registration, considering different modalities for microscopic images^29^. As a result, we used SURF^25^ features to compute the transformation in the pairwise registration.

Most of the computational load is allocated to pairwise registration. We found that 92.19% of the total stitching time belongs to pairwise registration. Therefore, developing a fast-stitching algorithm needs to have a fast and robust pairwise registration. To expedite the pairwise registration, we proposed two strategies that are fundamentally based on the fact that the microscopic images provide rich feature points because of the texture of the samples and cells.

**Figure 4:**
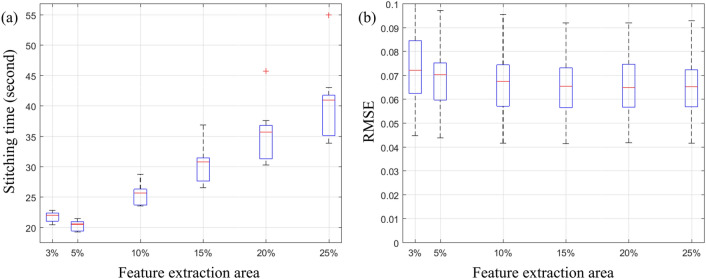
The impact of the size of the feature extraction area: (**a**) stitching time and (**b**) RMSE between the intensity of overlapping region considering different percentages of the width/height of tiles to extract features for Tak dataset images.

In the first strategy, SURF features were initially extracted and matched from a small portion of the overlapping region. If we were unable to compute the transformation, we then extracted and matched features from the entire overlapping region. To determine the smallest possible region for extracting features from tiles, we considered different percentages of the width/height of tiles, ranging from very small to the entire overlapping region. We examined this on the Tak dataset, taking into account 3% up to 25% of the width/height of tiles (25% is the overlap value of the Tak dataset, see Table 1), for feature extraction. Then, we calculated the average stitching time and the RMSE of the pixel intensity in the overlapping region of tiles as shown in Fig. 4. Limiting the feature extraction region up to 5% significantly reduced the stitching time (Fig. 4a), while the average RMSE between pairs of tiles (Fig. 4b) increased slightly. Moreover, this figure shows that decreasing the feature extraction area below 5% was not helpful since it increased both the stitching time and the RMSE value. Therefore, 5% of the width/height of the tile was considered the optimal area for feature extraction.

In the second strategy, we focused on extracting dominant features. The SURF feature extraction method computes the determinants of the Hessian matrix in space and scale and removes points with response values ​​below a threshold. The threshold value depends on the application and the image. In this study, the investigated image collections are from different modalities, and a threshold needs to be set for each modality. The threshold is chosen according to the distribution of Hessian determinant values ​​for different images. We analyzed the Hessian determinant values ​​and the number of features for 10 random bright-field images of the 026–01-91 image set from the Tak dataset. We noticed that the number of obtained features was quite large, but not all were useful. Additionally, the computational complexity of matching features between two tiles to find the transformation parameters directly depended on the number of features. As a result, choosing the strong features based on the determinant of the Hessian matrix could reduce the computational load of this step. For this purpose, we selected this threshold equal to 1000, considering 10% of the number of features. A similar analysis was performed on 10 different images of phase-contrast and fluorescence, and their threshold value was chosen to be equal to 1.

Moreover, the transformation model was assumed to be transitional because, firstly, the slide was imaged at a constant magnification in the WSI technique. Secondly, it was placed under the objective lens at a fixed angle. Thirdly, the changes in angle were so small that could be considered negligible. Overlapping tiles of the sample were acquired by moving the microscope motors only in two directions: horizontal and vertical. Therefore, in the computation of the transformation between two tiles, the scaling and rotation parameters were ignored, and only the displacement parameters in the horizontal (t_x_) and vertical (t_y_) directions were estimated.

We computed the parameters of the transformation model for the tile pairs using the MSAC algorithm (a variant of the RANSAC algorithm)^30,31^. The number of matched points was stored to compute the weight of the edges in the global alignment step. Computing the transformation between tiles lacking texture in the overlapping region is challenging because a few or no feature points can be detected or matched in this area, making the computation of the transformation for these tiles impossible or incorrect. For this reason, we filtered the computed transformations based on the movement range of the motor stages. Translations whose values are outside the valid range (overlapping region of tiles ± 2% of the width/height of tiles, accounting for uncertainty due to the imperfections of the automated microscope stages) were discarded. For a pair of tiles whose transformation was not computed or was discarded based on an invalid translation range, the transformation parameters and the number of matching points were set to NaN. Finally, the transformations between these tiles were estimated based on the computed transformations among the textured tiles. For the north direction, we replaced the invalid transformations with the average of transformations in that row. If that row had no valid transformations, the average of all valid north transformations was computed to replace the invalid transformations. Similarly, for the west direction, we replaced invalid transformations with the average of transformations in that column. If that column had no valid transformations, the average of all valid west transformations was computed to replace the invalid transformations.

### Global alignment

The main purpose of global alignment is to minimize error propagation by utilizing additional information obtained from the transformations computed between each tile and its four neighboring tiles, rather than the transformation computed between each tile and the subsequent neighboring tile. The chosen transformations used to compute the position of the tile have a direct impact on the overall quality of the final image^5^. At this step, the best match among tiles was selected to generate the final image. In the case of an image collection arranged in an M × N grid, the number of transformations computed during the pairwise alignment (2MN-(M + N)) was greater than the number of transformations required to determine the position of each tile in the final frame (MN-1). For this purpose, a weighted graph was constructed where each vertex represents a tile and is connected to its four neighboring tiles. The most important aspect of creating a graph, which controls the error propagation, is the weight of the edges. We computed the weight of each edge as the normalized inverse of the number of matched features between the two tiles connected to this edge (see Fig. 2b). This is because a higher number of matched features between pairs of tiles results in a more accurate and reliable transformation calculation. For non-informative pairs and filtered pairs in which the number of matched points was not defined, weights were specified by adding a constant number to the maximum computed weight to ensure that they had a minimal effect on the stitching result. After creating a graph, the Prim^32^ algorithm was used to build an MST and select the set of edges with the lowest total weight. The MST finds a subset of edges that form a tree including all the vertices of the graph and minimizing the total weight of the edges. Alternatively, Dijkstra's^33^ algorithm was used to build an SPT and choose the shortest route with the lowest total weight for each tile. Unlike the MST, which considers the total weight of edges in the tree, the SPT selects a path to the origin vertex for each vertex that has the lowest total weight. To minimize randomness, we chose an origin vertex where the sum of the path weights of all vertices to that vertex was minimum. Finally, the position of each tile in the final mosaic was computed based on constructed graphs. A frame was produced based on the number and dimensions of the tiles, and the tiles were placed in the final frame. To check the stitching errors and misalignment artifacts, no blending was applied to combine the tiles in the overlapping region, and the tiles were placed on top of each other in the overlapping region.

### Evaluation metrics

The results of stitching were assessed using objective criteria and visual inspection. The stitching time (including pairwise alignment and global alignment) and the average RMSE of the pixel intensity in the overlapping region of tile pairs based on the computed global positions were examined for objective evaluation. In addition, to evaluate the stitching accuracy of synthetic datasets, other objective criteria were computed besides the RMSE and stitching time. These included the centroid distance error (D_err_) between the tile’s centroid coordinates of the stitched and the ground truth images, the mean squared error (MSE), and the peak signal-to-noise ratio (PSNR) between the stitched and ground truth images.

## Results

The results of stitching using the FRMIS algorithm were compared with those of the MIST toolbox based on objective criteria and visual inspection. All codes were implemented using Matlab 2023a software on the operating system Intel® Core™ i7-1165G7 CPUs @ 2.80 GHz and 16 GB memory. To compare the stitching runtime, the FRMIS and MIST algorithms were executed on a single CPU core.

### Experimental datasets

The stitching time of different experimental datasets using the FRMIS and MIST algorithms is shown in Table 2. Stitching using the FRMIS method performed faster than the MIST method (up to 481% reduction for Tak, 259% for stem cell colony, and 282% for human colon datasets). Creating a graph based on MST or SPT did not significantly affect the speed of the stitching process since 0.01% of the computational time was related to global alignment and it controls error propagation.

**Table 2:** Stitching time (including pairwise alignment and global alignment) in seconds and an RMSE between the intensity of the overlapping region of image pairs for FRMIS and MIST methods on three different datasets.

Dataset	Modality	Data (# tiles)	Stitching time (sec.)			RMSE		
			FRMIS		MIST^9^	FRMIS		MIST^9^
			MST	SPT		MST	SPT	
Tak	Bright-field	026–01-91 (100)	23.16	**23.14**	139.49	5.67e-2	5.71e-2	**4.84e-2**
		051–04-80 (100)	**21.79**	**21.79**	132.57	6.85e-2	6.78e-2	**5.90e-2**
		156–01-86 (100)	**21.23**	**21.23**	132.08	7.63e-2	7.82e-2	**6.59e-2**
		194–01-70 (100)	**23.29**	**23.29**	135.62	8.45 e-2	8.11e-2	**7.01e-2**
		234–01-67 (100)	**22.28**	**22.28**	134.95	9.73e-2	9.74e-2	**8.49e-2**
		31–01 (100)	**24.89**	**24.89**	136.98	4.39e-2	4.37e-2	**4.03e-2**
		33–03 (100)	**23.15**	**23.15**	137.18	7.09e-2	6.96e-2	**6.27e-2**
		36–01 (100)	**23.14**	**23.14**	137.80	7.01e-2	6.95e-2	**6.22e-2**
		49–01 (100)	**24.93**	**24.93**	136.04	5.97e-2	6.01e-2	**5.50e-2**
		53–03 (100)	**27.22**	**27.22**	137.31	7.15e-2	**7.13e-2**	7.17e-2
Stem cell colony	Phase-contrast	level1 (100)	**11.07**	**11.07**	40.21	2.7e-3	2.7e-3	**2.6e-3**
		Phase (100)	**11.70**	**11.70**	43.64	**1.7e-3**	**1.7e-3**	**1.7e-3**
		Small_phase (25)	**2.87**	**2.87**	11.64	1.7e-3	**1.6e-3**	**1.6e-3**
	Fluorescence	level3 (552)	**74.43**	**74.43**	261.13	6.86e-4	6.82e-4	**6.75e-4**
		level2 (100)	**13.40**	**13.40**	45.79	5.24e-4	5.22e-4	**5.08e-4**
		Small_fluorescent (25)	**3.48**	**3.48**	11.08	**8.31e-4**	8.35e-4	8.32e-4
Human colon	Fluorescence	Human colon (609)	**56.30**	56.31	214.81	**7.47e-2**	7.53e-2	NaN

Best values are in bold.

**Figure 5:**
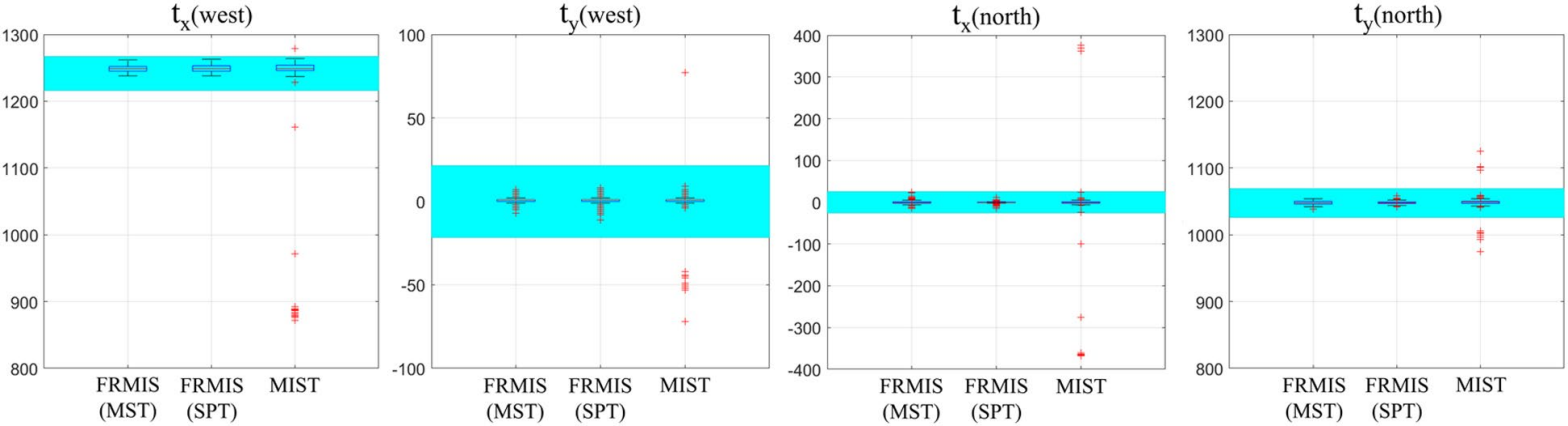
Distribution of computed translation parameters t_x_ and t_y_ in the west and north directions for the human colon dataset using the FRMIS (with MST and SPT graphs) and MIST (with known overlap value) methods. Note that the cyan boxes indicate the valid translation ranges.

The RMSE of the pixel intensity of tiles in the overlapping region was computed and presented in Table 2. The results indicated that the RMSE of the FRMIS method for the Tak dataset was higher (12.46%) compared to that of the MIST algorithm, but the discrepancy was too small to show any notable difference in terms of the visual quality of the stitched results produced by both methods. For the stem cell colony dataset in phase-contrast mode, the FRMIS method demonstrated the ability to stitch tiles with an error rate equivalent to that of the MIST algorithm. However, the stitching error of the FRMIS method was slightly higher (1.46%) compared to the MIST algorithm when applied in fluorescence mode. For the human colon dataset, the average RMSE of the MIST method could not be computed and was designated as NaN. In the stitched result, multiple adjacent tiles did not overlap because no transformations were computed for them, despite providing the overlap value of the human colon dataset to the MIST algorithm. To conduct a more comprehensive analysis of the findings from the human colon dataset using the FRMIS and MIST approaches, we proceeded to compare the computed translation parameters (t_x_ and t_y_) with the valid translation ranges depicted in Fig. 5. The valid translation range is determined by the overlapping region of tiles ± 2% of the width/height of tiles, taking into consideration the uncertainties caused by imperfections of the automated microscope stages. This figure highlights that MIST computed certain translation parameters (3.34%) that fell outside the valid range. The supplementary material, Fig. S1, visualizes the results reported in Table 2 to emphasize that the FRMIS method offers a rapid stitching algorithm for different modalities that performs comparably to the MIST algorithm.

**Figure 6:**
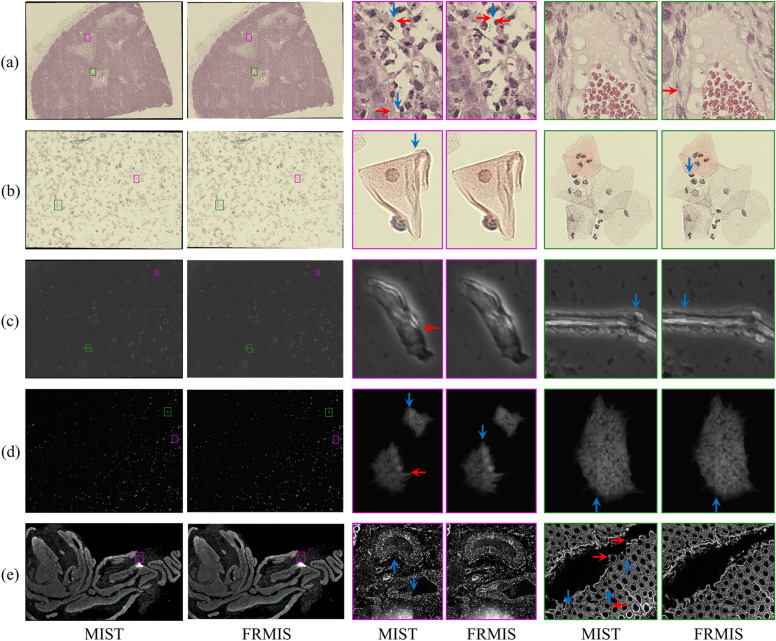
Stitched results of (**a**) the 36–01 image set from the Tak dataset, (**b**) the 026–01-91 image set from the Tak dataset, (**c**) the Phase image set from the stem cell colony dataset, (**d**) the Level3 image set from the stem cell colony dataset, and (e) the image set from human colon dataset using the FRMIS (with MST graph) and MIST methods. Red arrows denote horizontal errors and blue arrows denote vertical errors. Note: To check the stitching errors, no blending processing is used to combine the tiles in the overlapping region, and the tiles are placed on top of each other.

Additionally, a visual examination was conducted on the outcomes of the stitching algorithm. Figure 6 presents examples of the stitched results achieved through the utilization of the FRMIS (by the MST graph) and the MIST algorithms on the experimental datasets. The remaining stitched results can be found in the supplementary material, Figs. S2-S14. The red and blue arrows in Fig. 6 denote horizontal errors resulting from stitching with the northern neighbor, and vertical errors resulting from stitching with the western neighbor, respectively. It is important to note that no blending processing was employed to combine the tiles in the overlapping region to verify the stitching errors, and the tiles were simply placed on top of each other. When comparing the FRMIS algorithm with the MIST algorithm, it can be observed that the overall appearance of stitching results is similar. However, a comprehensive visual analysis of the results for the Tak and stem cell colony dataset (Fig. 6a–d) reveals that the FRMIS algorithm exhibits a lower number of occurring errors in certain areas compared to the MIST algorithm, while in other areas, the FRMIS algorithm shows a higher number of occurring errors. Inspecting the entire stitched image, we found that the number of occurring errors was similar and possibly smaller using the FRMIS algorithm compared with the MIST algorithm and the errors appeared at different locations. A closer investigation of the human colon dataset (Fig. 6e) showed that the MIST method had obvious stitching errors compared to the FRMIS approach.

**Figure 7:**
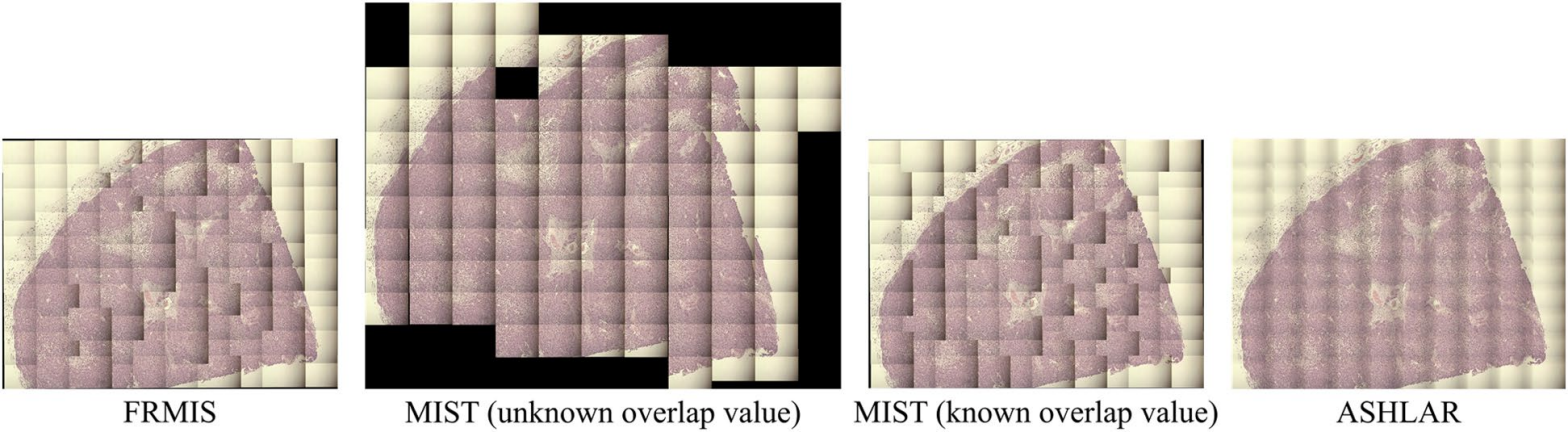
Stitched results of the 36–01 image set with shading from the Tak dataset using the FRMIS (with MST graph), MIST (with unknown overlap value), MIST (with known overlap value), and ASHLAR methods. Note: To check the stitching errors, no blending processing is used to combine the tiles in the overlapping region, and the tiles are placed on top of each other in the FRMIS and MIST methods, however, ASHLAR has used blending.

We also stitched tiles with uneven illumination from the original set of the Tak dataset using the FRMIS (by the MST graph), MIST, and ASHLAR methods, as shown in Fig. 7. It is worth mentioning that no blending processing was employed to combine the tiles in the overlapping region to verify stitching errors, and the tiles are placed on top of each other in the FRMIS and MIST methods. In contrast, the ASHLAR method utilized the blending technique. The findings indicate that all three algorithms are robust to shading when the overlap value is known, while the MIST algorithm failed to stitch images with shading when the overlap value is unknown. It is important to note that the overlap value is an option in the MIST algorithm, while it is required in the FRMIS and ASHLAR algorithms. Figure 7 displays the stitched results of the same image sets shown in Fig. 6a before the shading correction of the Tak dataset. The remaining stitched results can be accessed in the supplementary material, Figs. S15-S24.

### Synthetic dataset

We stitched the synthetic dataset using the FRMIS and MIST methods (supplementary material, Figs. S25–S36). First, we compared whether the dimensions of the mosaic image matched those of the ground truth image (supplementary material, Table S1 depicted the image dimensions. Note that the MIST algorithm output images had two rows and columns of extra black pixels). For objective evaluation, we computed the average RMSE and D_err_. In addition, for output images whose dimensions were equal to the ground truth image (by removing the extra pixels of the MIST output), we measured the MSE and PSNR of the stitched images and the corresponding ground truth images. For all investigated ICIAR image sets, the RMSE between image pairs, D_err_, and MSE were zero, and the PSNR values were infinite. As a result, stitching of these images using all methods was error-free. Analyzing the error metrics of the USAF image sets (supplementary material, Table S2) revealed that the mosaic constructed by the FRMIS method had less error compared to the MIST. Supplementary material, Table S3 shows the stitching time of ICIAR image sets and USAF slides using FRMIS and MIST algorithms. Using the proposed method, stitching of the ICIAR and USAF image sets ran faster than the MIST method (up to 311% reduction for ICIAR image sets and 484% reduction for USAF slides).

## Discussion

We present the results of the proposed FRMIS algorithm and compare them to the existing stitching algorithm. Various open-source toolboxes for microscopic image stitching, including Terastitcher^14^, istitch^8^, FijiIs^19^, ASHLAR^11^, and MIST^9^, are available. The authors in^11^ have determined that ASHLAR and MIST exhibit comparable accuracy in stitching, with MIST demonstrating a slightly faster stitching speed than ASHLAR. Furthermore, the MIST algorithm can be applied to different image modalities. We have compared the FRMIS algorithm with the MIST toolbox as it outperforms other toolboxes in terms of speed and/or error rate in stitching tiles to generate a whole-slide image. Objective assessment of the experimental datasets (Table 2) highlights that the FRMIS algorithm with MST graph resembled the performance of the MIST algorithm in the case of the Tak and stem cell colony datasets and showed better performance in the case of the human colon dataset with significant improvement on the stitching time (481% faster for Tak, 259% for stem cell colony, and 282% for human colon datasets). Two main reasons are contributing to the reduction of stitching time: restricting the feature extraction area and focusing on the dominant features in the pairwise registration, as discussed in detail in the method section. That made the algorithm more efficient, as 92.19% of the computational load is attributed to the pairwise alignment. Additionally, it reduced the misalignment that might occur by matching duplicated features in tiles with repetitive textures.

As indicated in the results presented in Table 2, the MIST stitching results had a lower RMSE compared to the FRMIS algorithm. However, visual inspections showed that the MIST algorithm produced more artifacts compared to the FRMIS method (Fig. 6). The reason for the lower RMSE in the MIST algorithm is that it employed a technique that optimizes the computed transformations by applying constrained hill climbing to the NCC values after pairwise registration. We incorporated this approach into the FRMIS algorithm and achieved the same level of accuracy as MIST, while almost halving our stitching time compared to the MIST algorithm (see supplementary material, Table S4). It is recommended that future research focuses on developing an optimized method that minimizes error with minimal impact on computational time.

A closer examination of the human colon dataset (Fig. 6e) showed that the MIST method had obvious stitching errors compared to the FRMIS approach. This was also reflected in the NaN RMSE value, which indicates that multiple adjacent tiles did not overlap in the stitched result using the MIST method even if the overlap value is given. The reason is that the MIST algorithm used phase correlation for the pairwise registration. This method required a large overlap region to perform properly^34^, but the FRMIS algorithm did not have this limitation. ASHLAR used sub-pixel precision phase correlation for stitching, and it has overcome this issue at the expense of a slower stitching process compared to MIST.

Moreover, our algorithm is robust as it does not depend on the illumination of the tile (Fig. 7). Uneven illumination is a common problem in various microscopic imaging techniques such as bright-field. It results in intensity inhomogeneity and leads to errors and artifacts in the stitching algorithms particularly those that are intensity-based such as MIST and ASHLAR. The feature-based approaches like FRMIS are invariant to rotation, translation, and brightness variance that occur in optical microscopic images.

The SURF features have been utilized by Qidway et al.^16^ and Yang et al.^17^ to stitch microscopic images using the entire overlapping region of tiles. Their algorithms were specifically applied to a limited number of tiles from bright-field modality, neglecting global alignment and generation of the whole-slide image. We speeded up the stitching algorithm by using dominant feature points extracted only from a small portion of the overlapping region. Limiting the feature extraction region also prevents errors in feature matching caused by repeating patterns in tiles. Moreover, considering the number of matched feature points in creating graphs provides less misalignment in the final stitched image.

## Conclusion

In this study, we have developed a fast and robust automatic stitching algorithm to generate a consistent whole-slide image. The proposed FRMIS approach utilizes dominant SURF features from a small part of the overlapping region to achieve pairwise registration and take into account the number of matched features in the global alignment. This algorithm reduces computational time and misalignment. We have shown that the FRMIS algorithm is superior to the existing stitching algorithms in terms of speed and accuracy in stitched images from different image modalities, including bright-field, phase-contrast, and fluorescence.

### Supplementary Information

Supplementary Information.

## Data Availability

Data availability. The Tak dataset which was included in the published article^20^, can be accessed at https://github.com/pair-kopti/Shading-correction. Similarly, the human colon dataset is available through Sage Synapse at https://dx.doi.org/10.7303/syn25826362 (a free account is required to access the data). Additionally, the images of the stem cell colony dataset can be downloaded from https://isg.nist.gov/BII_2015/webPages/pages/stitching/Stitching.html and the images of the ICIAR can be downloaded from https://iciar2018-challenge.grand-challenge.org/Dataset/.

## Code availability

https://github.com/labCOI/FRMIS.
