# Supplementary material for: Fast and robust feature-based stitching algorithm for microscopic images
Source: Sci Rep. 2024 Jun 10;14:13304. doi: 10.1038/s41598-024-61970-y (PMC11164998; doi:10.1038/s41598-024-61970-y)
Supplement: Supplementary file 1 — Supplementary Information. [file 41598_2024_61970_MOESM1_ESM.pdf]

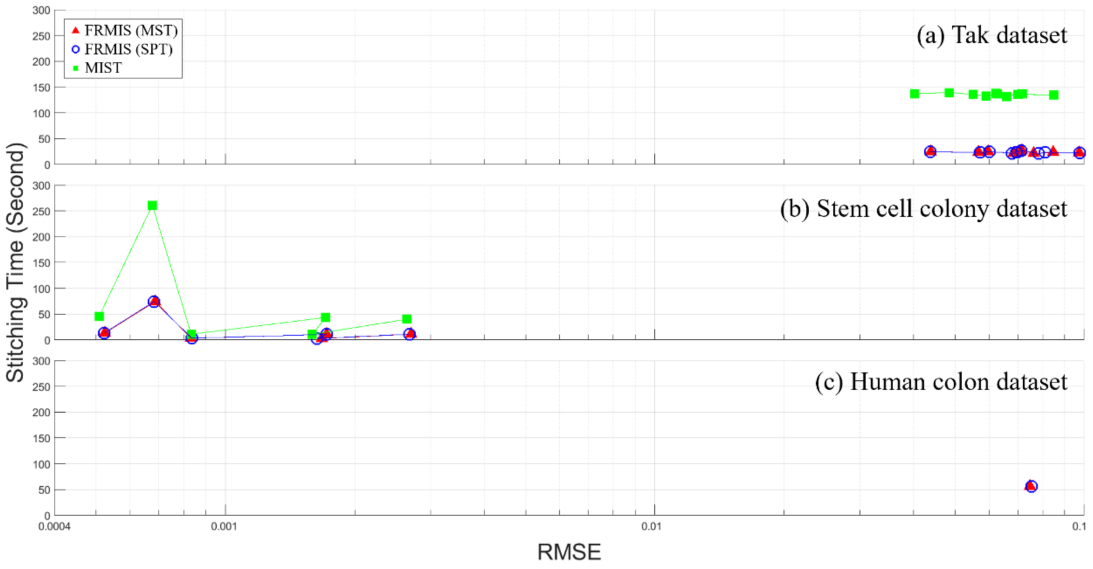

Figure S1. Diagram of stitching time and an average of RMSE of the pixel intensity in the overlapping region of two adjacent tiles using the proposed FRMIS and MIST methods. (a) Tak dataset, (b) Stem cell colony dataset, and (c) Human colon dataset.

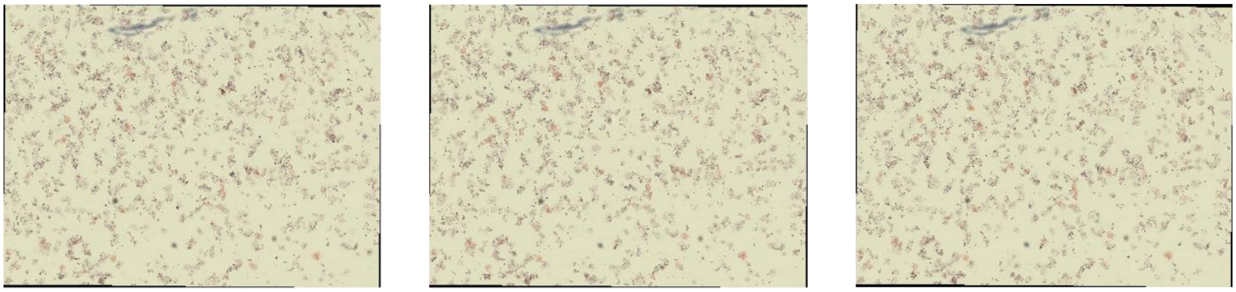

(a) FRMIS-MST (b) FRMIS-SPT (c) MIST

Figure S2. Stitching results of the 026-01-91 image set from the Tak dataset using (a-b) FRMIS and (c) MIST methods.

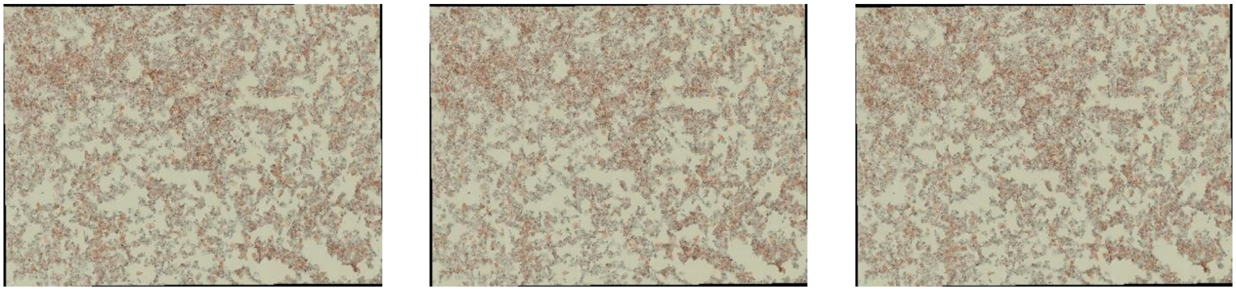

(a) FRMIS-MST (b) FRMIS-SPT (c) MIST

Figure S3. Stitching results of the 051-04-80 image set from the Tak dataset using (a-b) FRMIS and (c) MIST methods.

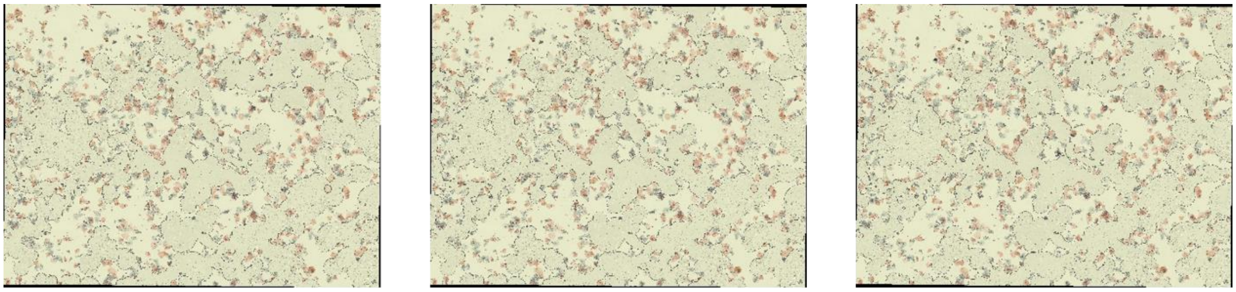

(a) FRMIS-MST (b) FRMIS-SPT (c) MIST

Figure S4. Stitching results of the 156-01-86 image set from the Tak dataset using (a-b) FRMIS and (c) MIST methods.

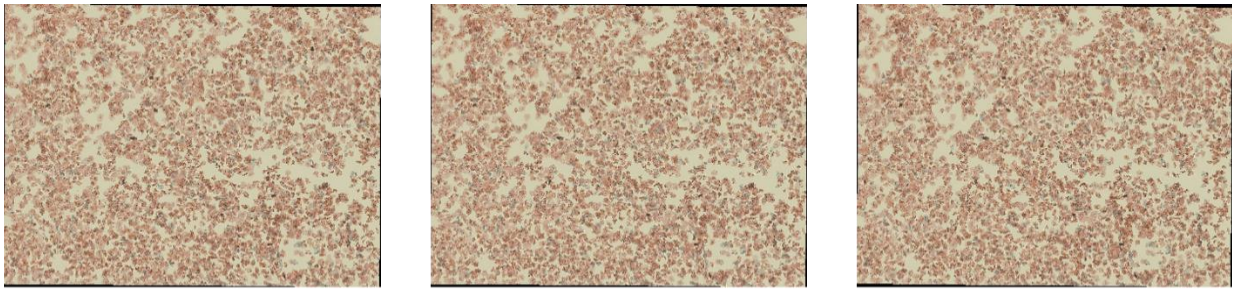

(a) FRMIS-MST (b) FRMIS-SPT (c) MIST

Figure S5. Stitching results of the 194-01-70 image set from the Tak dataset using (a-b) FRMIS and (c) MIST methods.

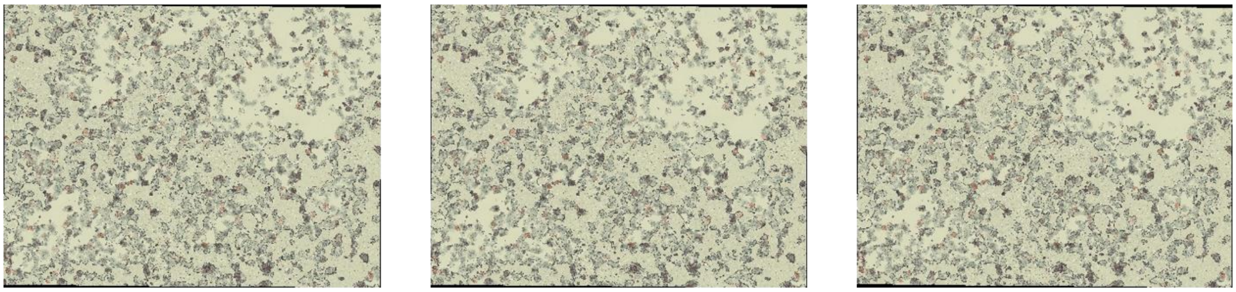

(a) FRMIS-MST (b) FRMIS-SPT (c) MIST

Figure S6. Stitching results of the 234-01-67 image set from the Tak dataset using (a-b) FRMIS and (c) MIST methods.

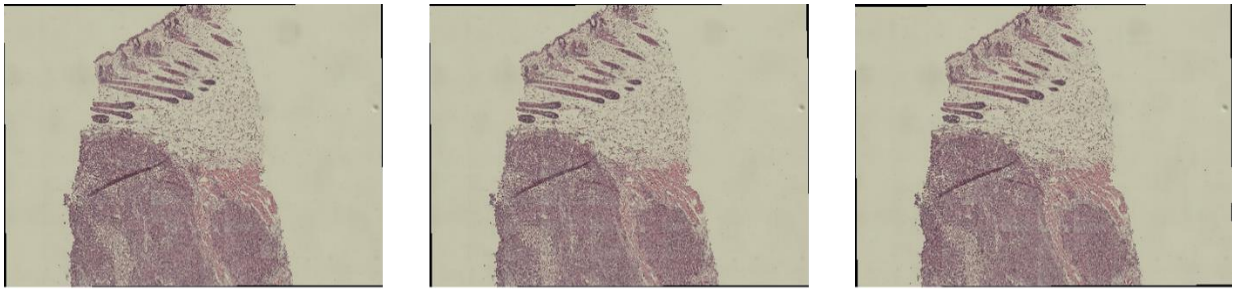

(a) FRMIS-MST (b) FRMIS-SPT (c) MIST

Figure S7. Stitching results of the 31-01 image set from the Tak dataset using (a-b) FRMIS and (c) MIST methods.

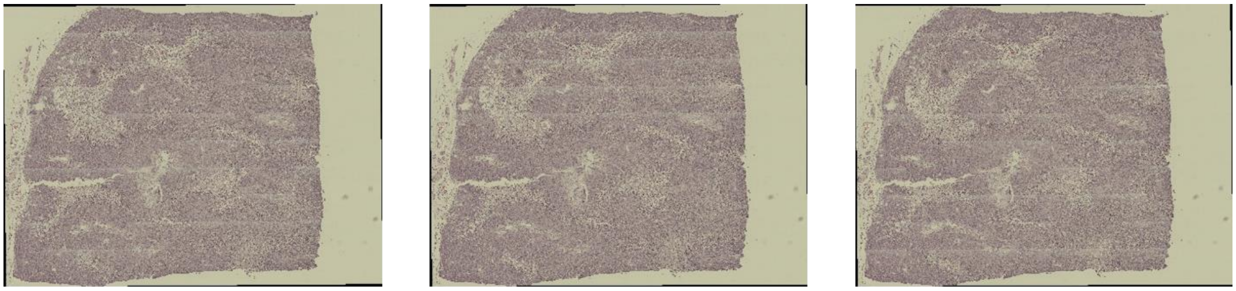

(a) FRMIS-MST (b) FRMIS-SPT (c) MIST

Figure S8. Stitching results of the 33-03 image set from the Tak dataset using (a-b) FRMIS and (c) MIST methods.

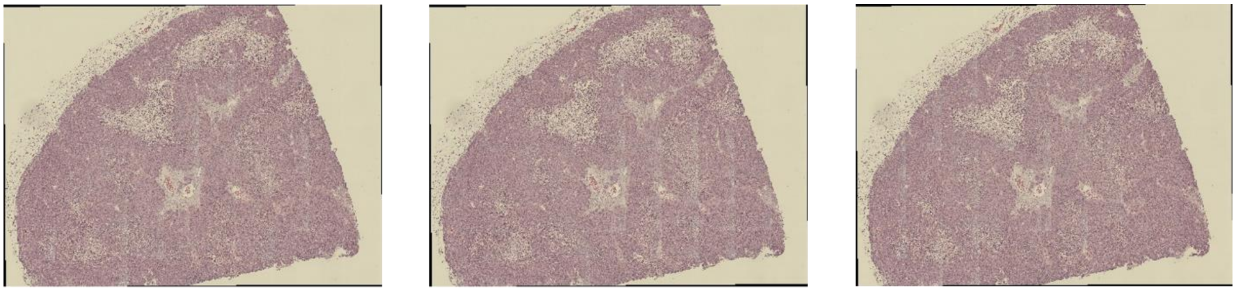

(a) FRMIS-MST (b) FRMIS-SPT (c) MIST

Figure S9. Stitching results of the 36-01 image set from the Tak dataset using (a-b) FRMIS and (c) MIST methods.

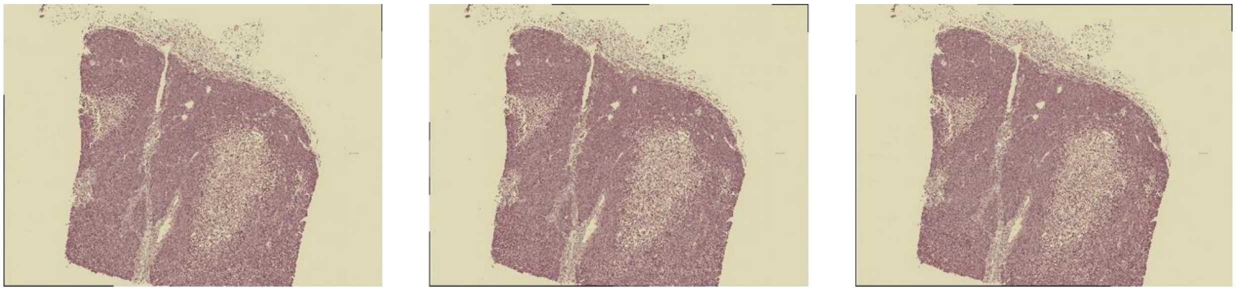

(a) FRMIS-MST (b) FRMIS-SPT (c) MIST

Figure S10. Stitching results of the 49-01 image set from the Tak dataset using (a-b) FRMIS and (c) MIST methods.

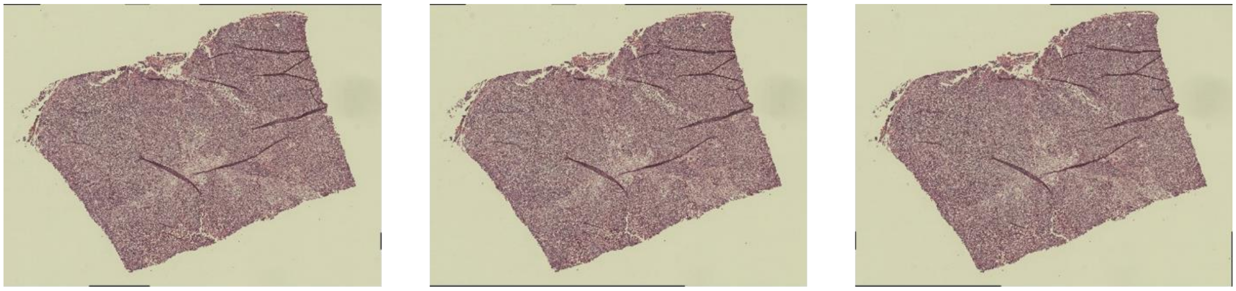

(a) FRMIS-MST (b) FRMIS-SPT (c) MIST

Figure S11. Stitching results the of 53-03 image set from the Tak dataset using (a-b) FRMIS and (c) MIST methods.

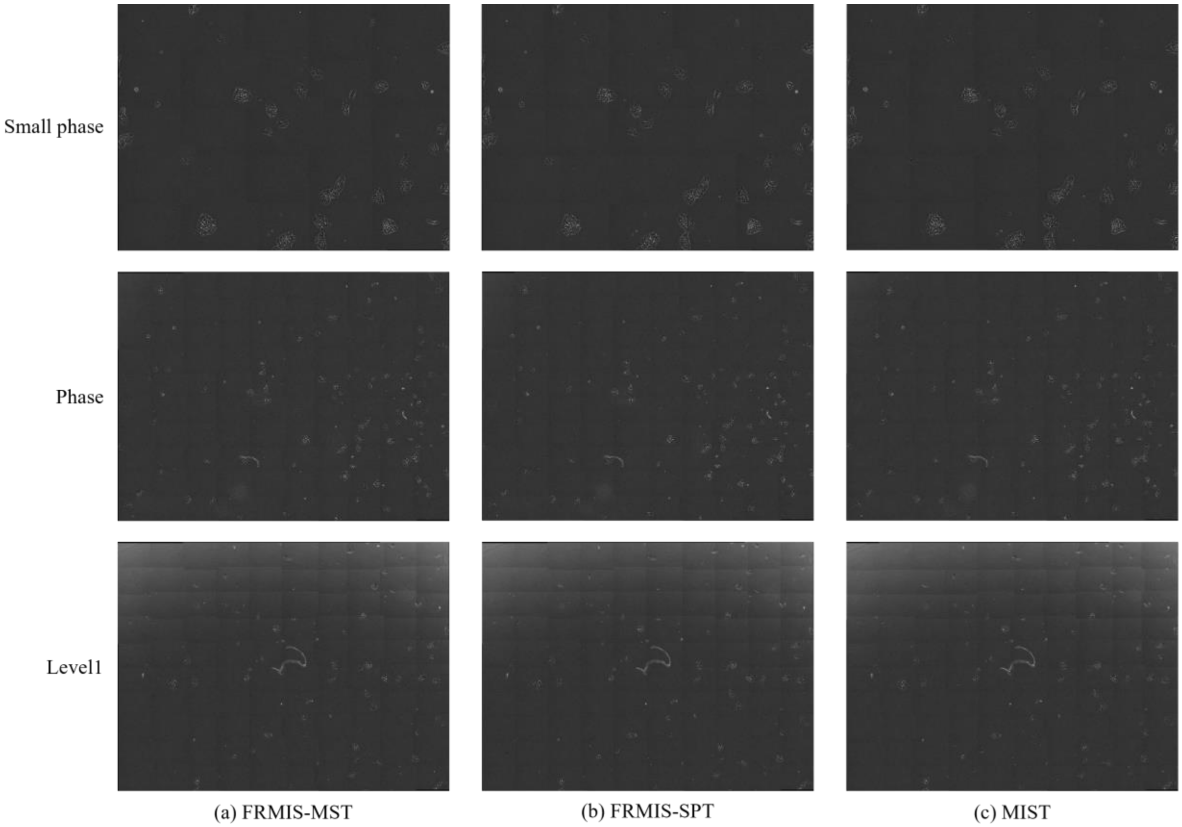

Figure S12. Stitching results of the phase-contrast image sets from the Stem cell colony dataset using (a-b) FRMIS and (c) MIST methods.

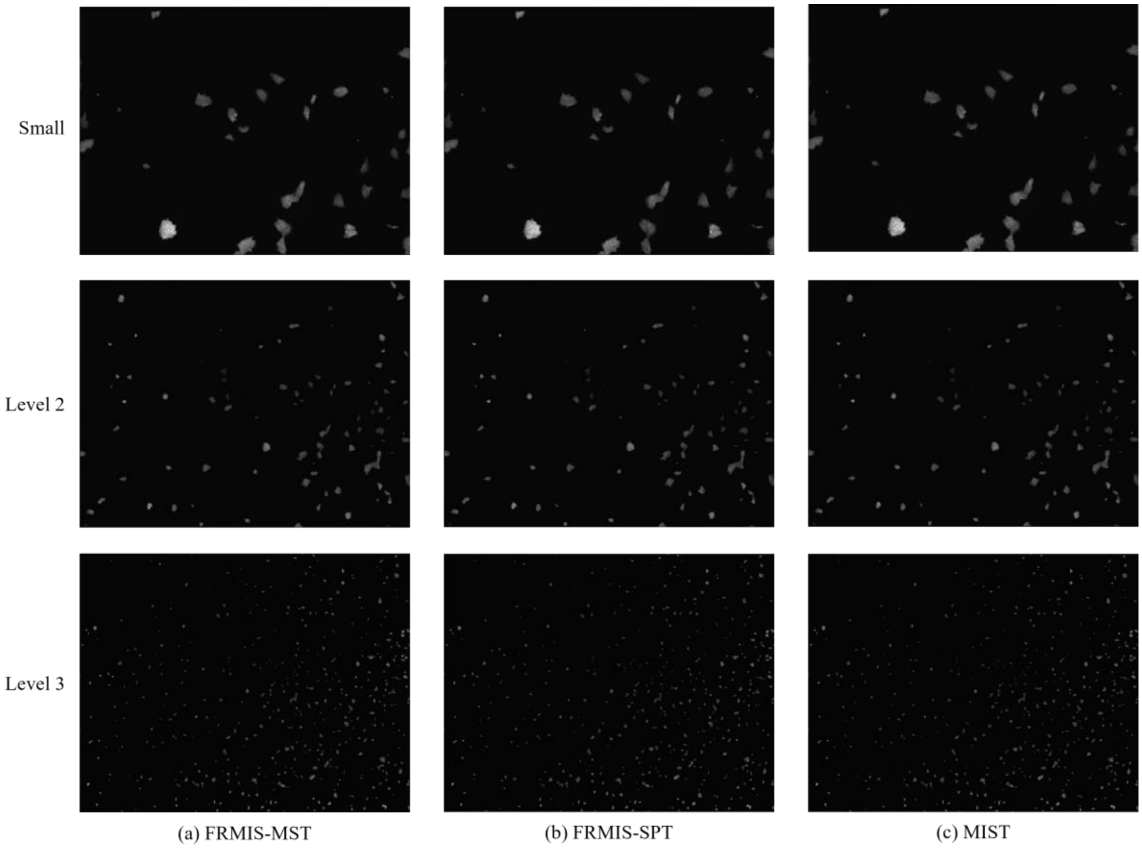

Figure S13. Stitching results of the fluorescent-contrast image sets from the Stem cell colony dataset using (a-b) FRMIS and (c) MIST methods.

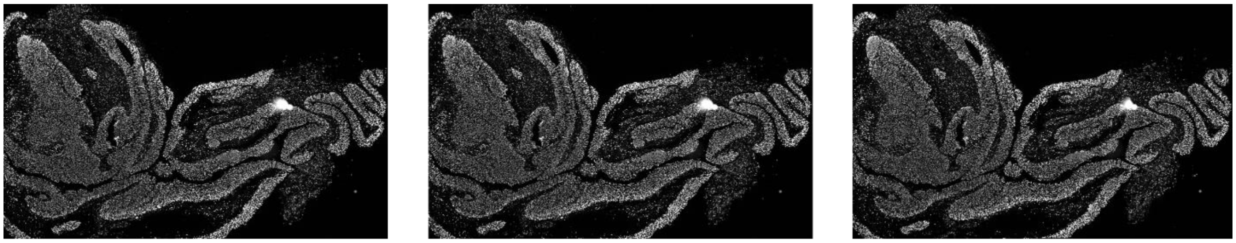

(a) FRMIS-MST (b) FRMIS-SPT (c) MIST

Figure S14. Stitching results of the image set from the Human colon dataset using (a-b) FRMIS and (c) MIST methods.

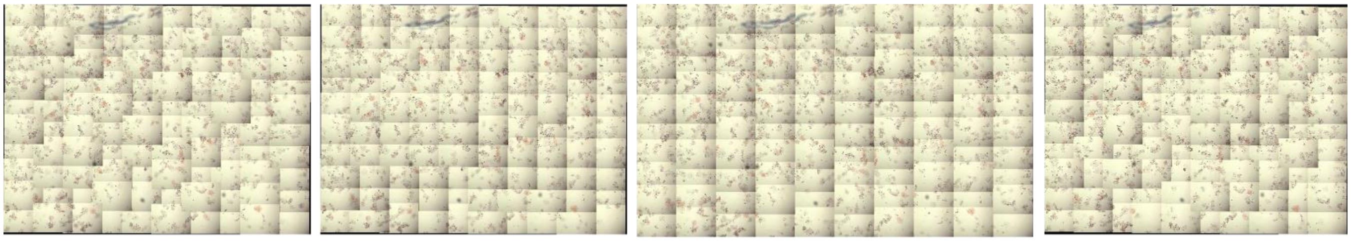

(a) FRMIS-MST (b) FRMIS-SPT (c) MIST-unknown overlap value (d) MIST-known overlap value

Figure S15. Stitching results of the 026-01-91 image set with shading from the Tak dataset using (a-b) FRMIS and (c-d) MIST methods.

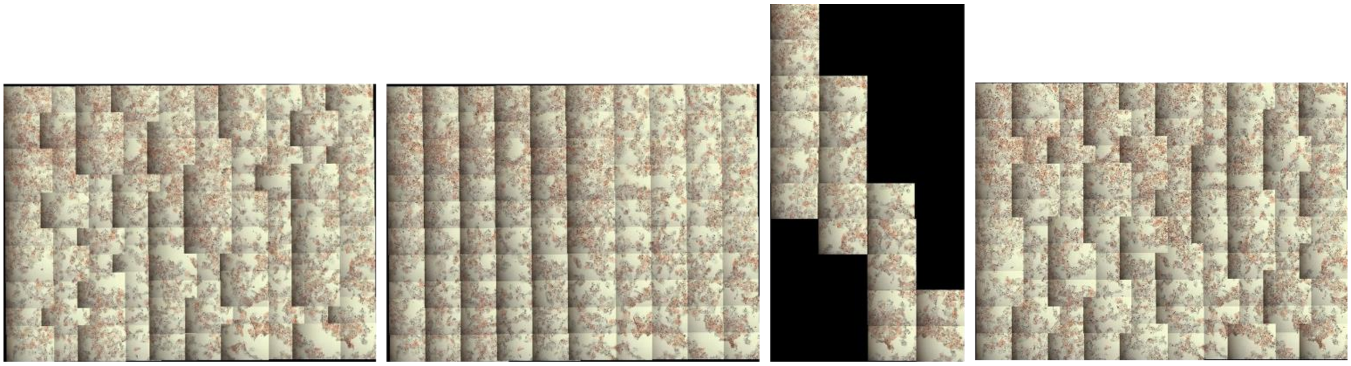

(a) FRMIS-MST (b) FRMIS-SPT (c) MIST unknown overlap value (d) MIST-known overlap value

Figure S16. Stitching results of the 051-04-80 image set with shading from the Tak dataset using (a-b) FRMIS and (c-d) MIST methods.

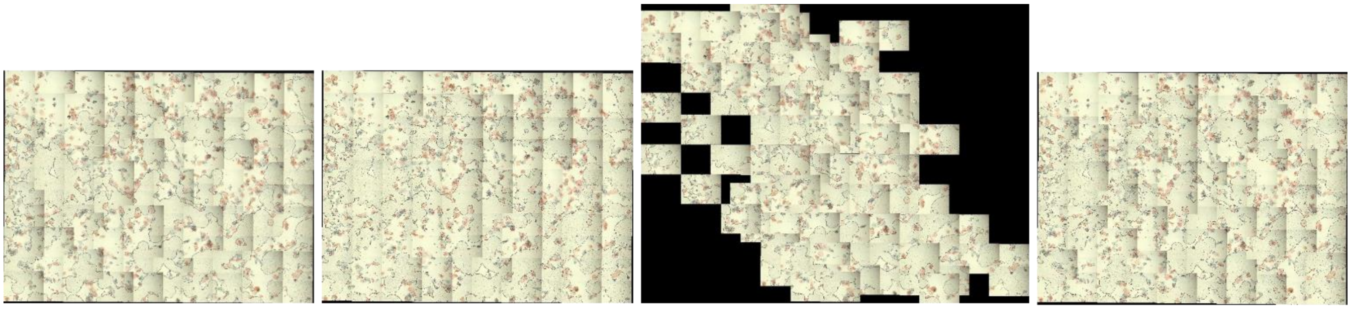

(a) FRMIS-MST (b) FRMIS-SPT (c) MIST-unknown overlap value (d) MIST-known overlap value

Figure S17. Stitching results of the 156-01-86 image set with shading from the Tak dataset using (a-b) FRMIS and (c-d) MIST methods.

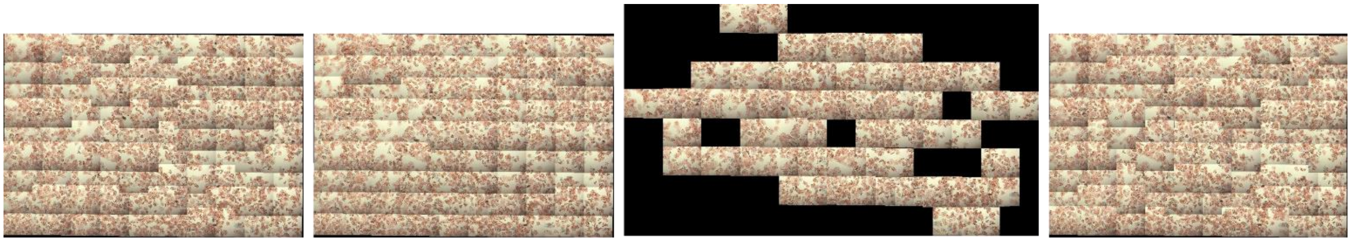

(a) FRMIS-MST (b) FRMIS-SPT (c) MIST-unknown overlap value (d) MIST-known overlap value

Figure S18. Stitching results of the 194-01-70 image set with shading from the Tak dataset using (a-b) FRMIS and (c-d) MIST methods.

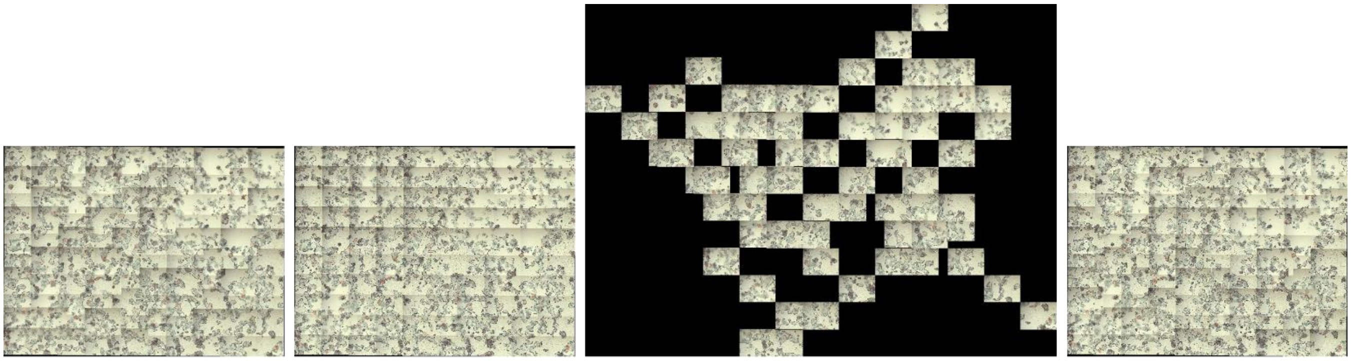

(a) FRMIS-MST (b) FRMIS-SPT (c) MIST-unknown overlap value (d) MIST-known overlap value

Figure S19. Stitching results of the 234-01-67 image set with shading from the Tak dataset using (a-b) FRMIS and (c-d) MIST methods.

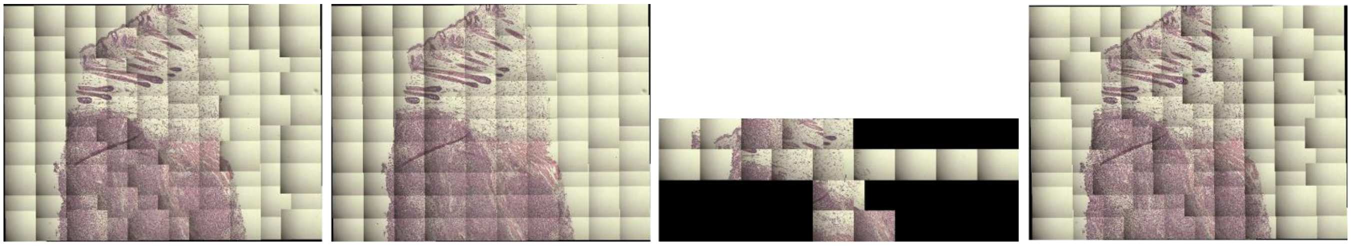

(a) FRMIS-MST (b) FRMIS-SPT (c) MIST-unknown overlap value (d) MIST-known overlap value

Figure S20. Stitching results of the 31-01 image set with shading from the Tak dataset using (a-b) FRMIS and (c-d) MIST methods.

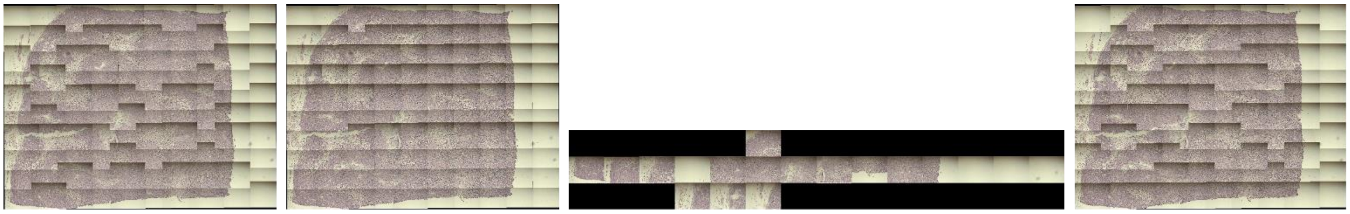

(a) FRMIS-MST (b) FRMIS-SPT (c) MIST-unknown overlap value (d) MIST-known overlap value

Figure S21. Stitching results of the 33-03 image set with shading from the Tak dataset using (a-b) FRMIS and (c-d) MIST methods.

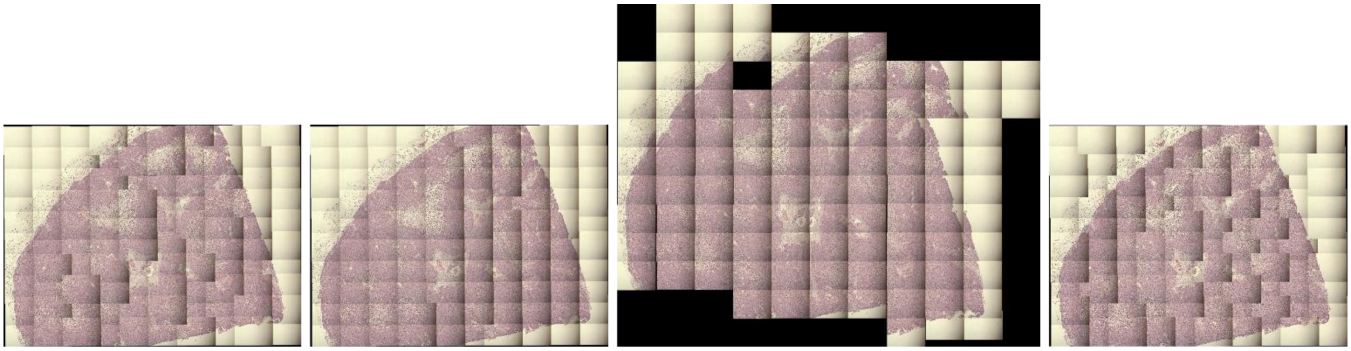

(a) FRMIS-MST (b) FRMIS-SPT (c) MIST-unknown overlap value (d) MIST-known overlap value

Figure S22. Stitching results of the 36-01 image set with shading from the Tak dataset using (a-b) FRMIS and (c-d) MIST methods.

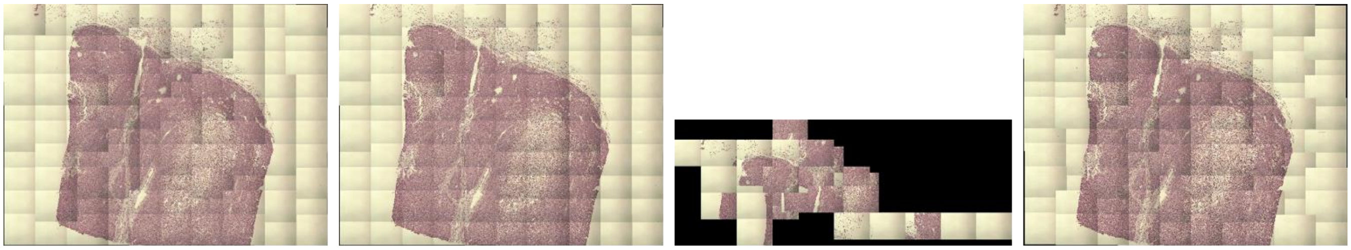

(a) FRMIS-MST (b) FRMIS-SPT (c) MIST-unknown overlap value (d) MIST-known overlap value

Figure S23. Stitching results of the 49-01 image set with shading from the Tak dataset using (a-b) FRMIS and (c-d) MIST methods.

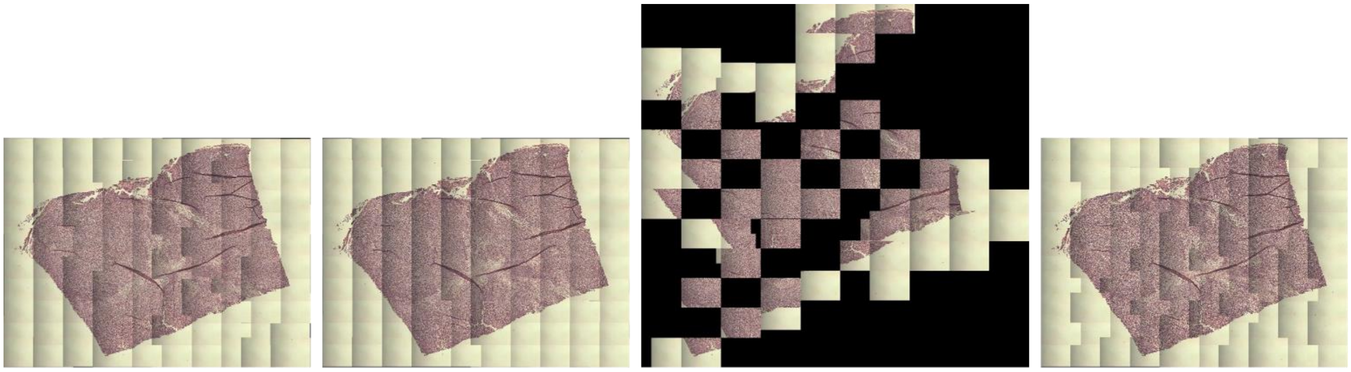

(a) FRMIS-MST (b) FRMIS-SPT (c) MIST-unknown overlap value (d) MIST-known overlap value

Figure S24. Stitching results of the 53-03 image set with shading from the Tak dataset using (a-b) FRMIS and (c-d) MIST methods.

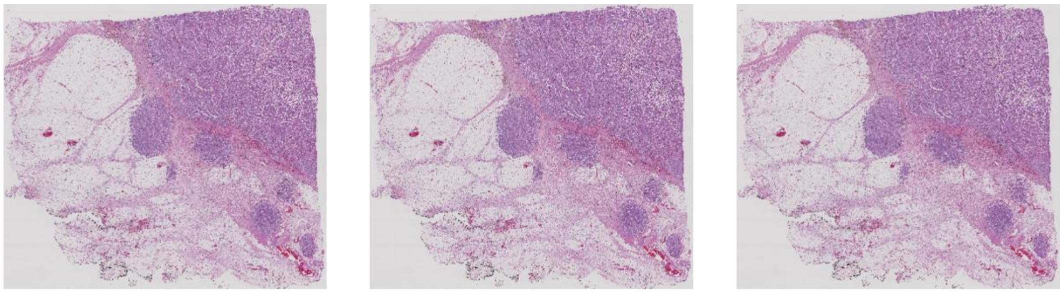

(a) FRMIS-MST (b) FRMIS-SPT (c) MIST

Figure S25. Stitching results of the Test1 image set from the ICIAR dataset using (a-b) FRMIS and (c) MIST methods.

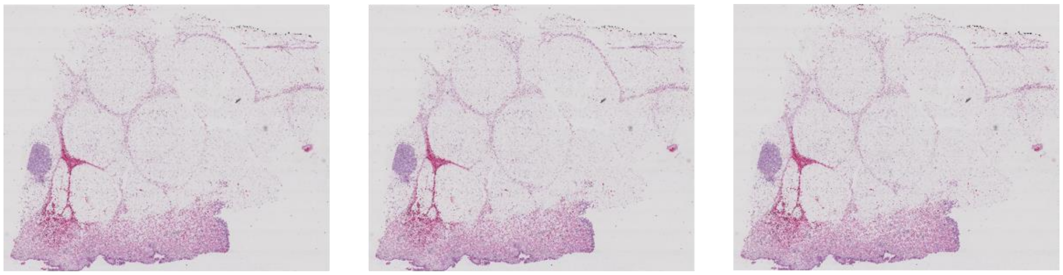

(a) FRMIS-MST (b) FRMIS-SPT (c) MIST

Figure S26. Stitching results of the Test2 image set from the ICIAR dataset using (a-b) FRMIS and (c) MIST methods.

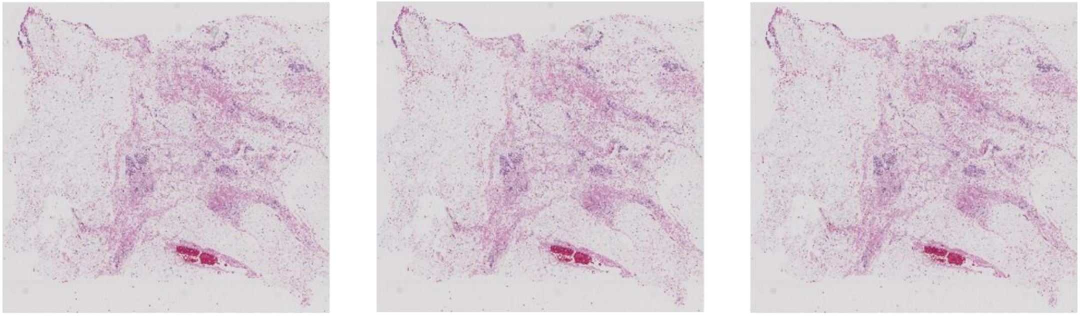

(a) FRMIS-MST (b) FRMIS-SPT (c) MIST

Figure S27. Stitching results of the Test3 image set from the ICIAR dataset using (a-b) FRMIS and (c) MIST methods.

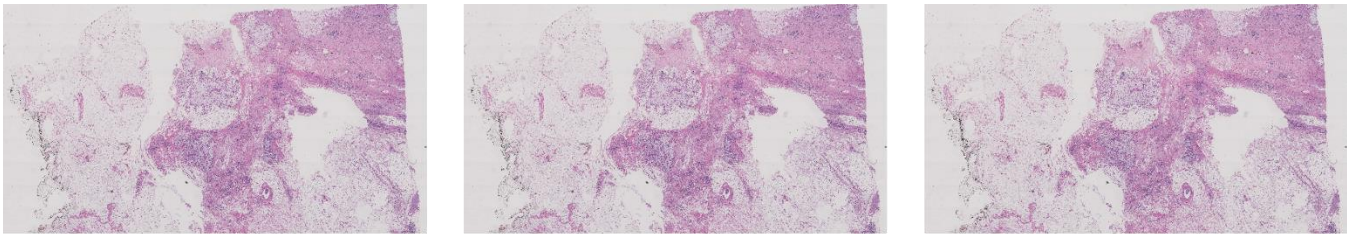

(a) FRMIS-MST (b) FRMIS-SPT (c) MIST

Figure S28. Stitching results of the Test4 image set from the ICIAR dataset using (a-b) FRMIS and (c) MIST methods.

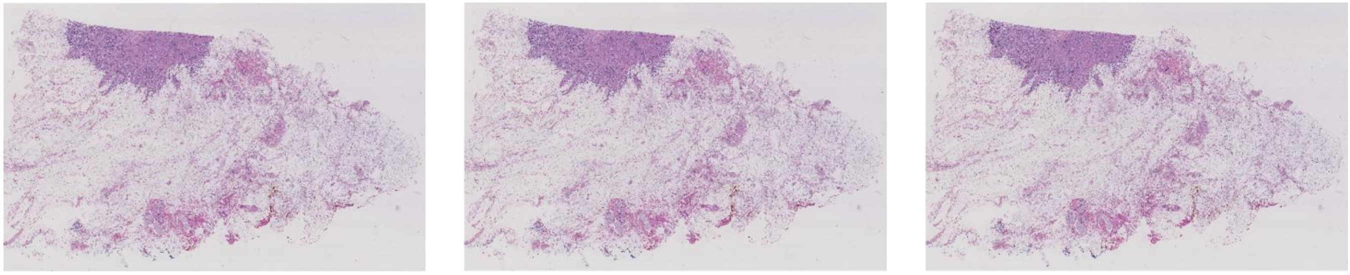

(a) FRMIS-MST (b) FRMIS-SPT (c) MIST

Figure S29. Stitching results of the Test5 image set from the ICIAR dataset using (a-b) FRMIS and (c) MIST methods.

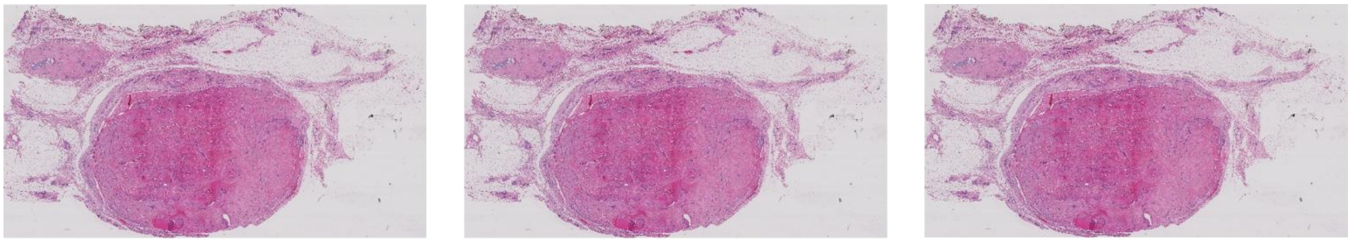

(a) FRMIS-MST (b) FRMIS-SPT (c) MIST

Figure S30. Stitching results of the Test6 image set from the ICIAR dataset using (a-b) FRMIS and (c) MIST methods.

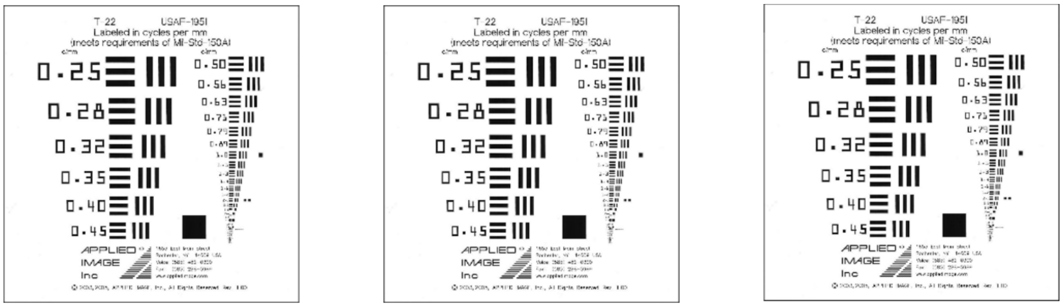

(a) FRMIS-MST (b) FRMIS-SPT (c) MIST

Figure S31. Stitching results of the T-22-P-CG image set from the USAF dataset using (a-b) FRMIS and (c) MIST methods.

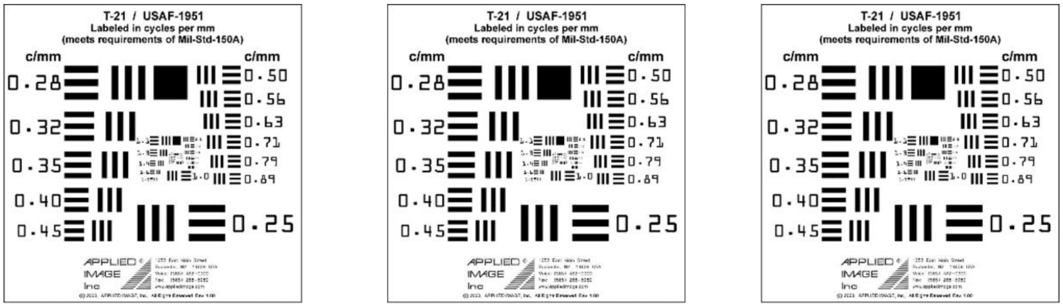

(a) FRMIS-MST (b) FRMIS-SPT (c) MIST

Figure S32. Stitching results of the T-21-P-CG image set from the USAF dataset using (a-b) FRMIS and (c) MIST methods.

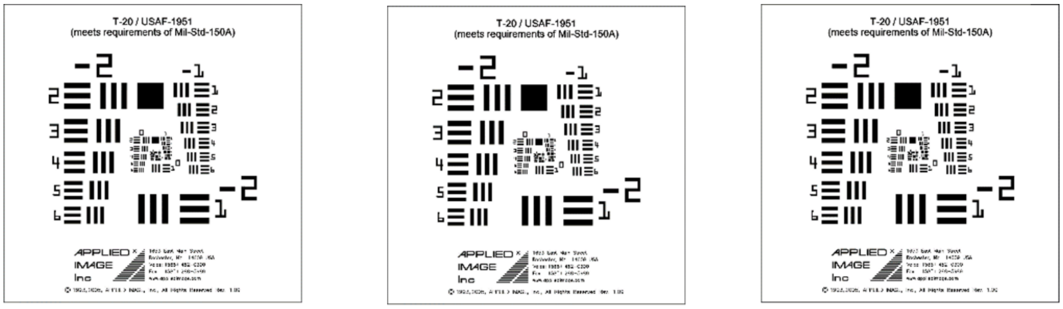

(a) FRMIS-MST (b) FRMIS-SPT (c) MIST

Figure S33. Stitching results of the T-20-P-CG image set from the USAF dataset using (a-b) FRMIS and (c) MIST methods.

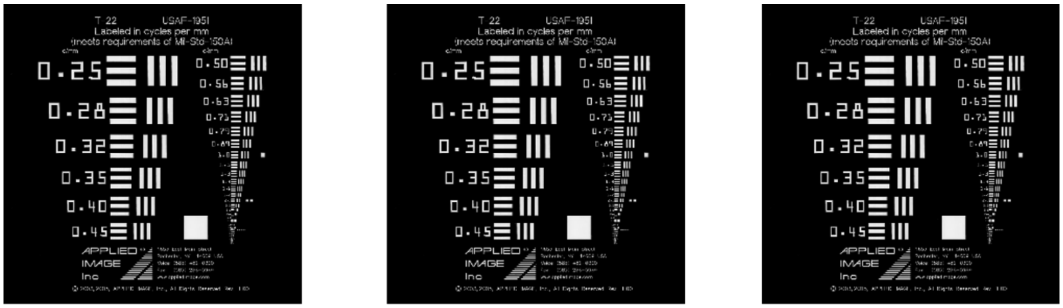

(a) FRMIS-MST (b) FRMIS-SPT (c) MIST

Figure S34. Stitching results of the T-22-2-N-CG image set from the USAF dataset using (a-b) FRMIS and (c) MIST methods.

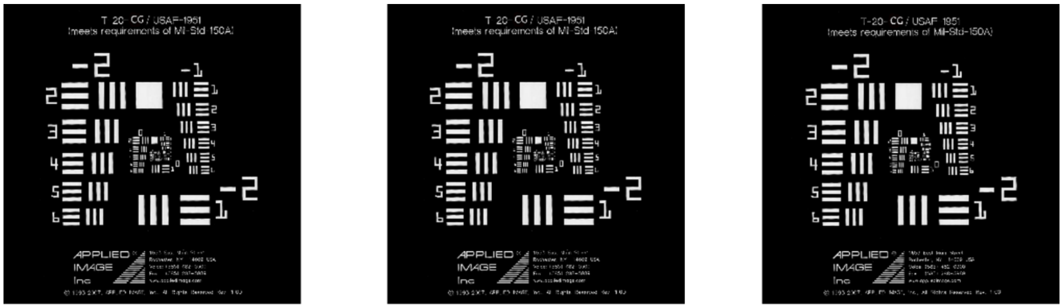

(a) FRMIS-MST (b) FRMIS-SPT (c) MIST

Figure S35. Stitching results of the T-20-N-CG image set from the USAF dataset using (a-b) FRMIS and (c) MIST methods.

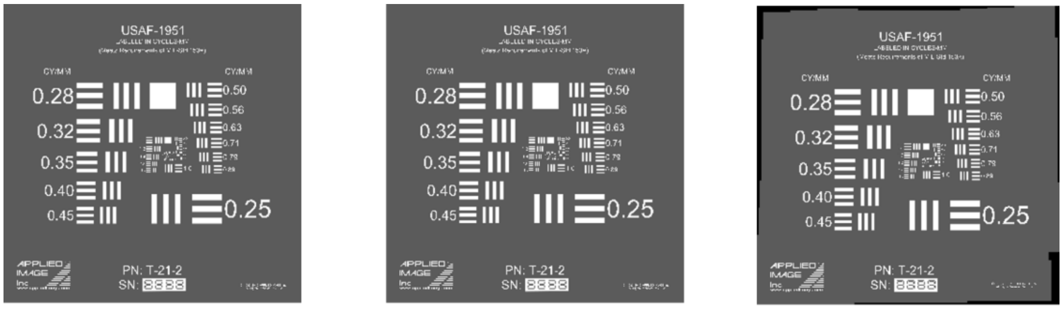

(a) FRMIS-MST (b) FRMIS-SPT (c) MIST

Figure S36. Stitching results of the T-21-2-N-OP image set from the USAF dataset using (a-b) FRMIS and (c) MIST methods.

Table S1. Dimension of ground-truth and stitched images (pixel), ICIAR and USAF dataset. Note. The MIST algorithm output images have two rows and columns of extra black pixels

Dataset	Image Set	Ground Truth size	FRMIS		MIST [9]
			MST	SPT	
ICIAR	Test1	**10663×9397**	**10663×9397**	**10663×9397**	10665×9399
	Test2	**13781×11222**	**13781×11222**	**13781×11222**	13783×11224
	Test3	**8797×8343**	**8797×8343**	**8797×8343**	8799×8345
	Test4	**15738×8530**	**15738×8530**	**15738×8530**	15740×8532
	Test5	**14262×9061**	**14262×9061**	**14262×9061**	14264×9063
	Test6	**12710×6993**	**12710×6993**	**12710×6993**	12712×6995
USAF	T-22-P-CG	**9504×9528**	**9504×9528**	**9504×9528**	9506×9530
	T-21-P-CG	**9600×9600**	**9600×9600**	**9600×9600**	9602×9602
	T-20-P-CG	**9600×9600**	9599×9603	9599×9603	9602×9597
	T-22-2-N-CG	**9504×9528**	**9504×9528**	**9504×9528**	9509×9533
	T-20-N-CG	**9432×9488**	9434×9488	9434×9488	9475×9531
	T-21-2-N-OP	**12000×12000**	12001×11995	12001×12000	12190×12002

Table S2. Stitching error of USAF dataset

Image Set	Error metric	FRMIS		MIST [9]
		MST	SPT	
T-22-P-CG	MSE	**4.76×10^{-5}**	**4.76×10^{-5}**	9.38×10^{-5}
	PSNR	**43.23**	**43.23**	40.2786
	D_{err}	**0**	**0**	0.41
	RMSE	**0**	**0**	4.64×10^{-4}
T-21-P-CG	MSE	**1.90×10^{-5}**	**1.90×10^{-5}**	1.99×10^{-5}
	PSNR	**47.22**	**47.22**	47.01
	D_{err}	**0**	**0**	1.92
	RMSE	**0**	**0**	1.4×10^{-3}
T-20-P-CG	MSE	-	-	-
	PSNR	-	-	-
	D_{err}	1.46	**1.44**	9.16
	RMSE	1.6×10^{-3}	**6.45×10^{-4}**	5.67×10^{-4}
T-22-2-N-CG	MSE	**3.09×10^{-5}**	**3.09×10^{-5}**	2.8×10^{-3}
	PSNR	**45.10**	**45.10**	25.50
	D_{err}	**0**	**0**	3.95
	RMSE	**0**	**0**	**0**
T-20-N-CG	MSE	-	-	-
	PSNR	-	-	-
	D_{err}	**1**	**1**	52.21
	RMSE	8.59×10^{-6}	8.59×10^{-6}	**0**
T-21-2-N-OP	MSE	-	-	-
	PSNR	-	-	-
	D_{err}	1.93	**1.05**	221.79
	RMSE	**2.86×10^{-4}**	**2.86×10^{-4}**	4.82×10^{-4}

Table S3. Stitching time (in seconds) of ICIAR and USAF dataset

Dataset	Image Set	FRMIS		MIST [9]
		MST	SPT	
ICIAR	Test1	15.62	15.62	61.78
	Test2	22.07	22.07	105.67
	Test3	11.71	11.71	43.61
	Test4	18.80	18.80	80.59
	Test5	19.55	19.55	78.23
	Test6	14.05	14.05	54.86
USAF	T-22-P-CG	18.88	18.88	90.54
	T-21-P-CG	14.61	14.61	110.59
	T-20-P-CG	21.80	21.80	218.46
	T-22-2-N-CG	15.38	15.38	74.66
	T-20-N-CG	13.95	13.95	56.19
	T-21-2-N-OP	14.84	14.84	56.28

Table S4. Stitching time (including pairwise alignment and global alignment) in seconds and an average RMSE error between the intensity of the overlapping region of image pairs for the proposed FRMIS method with optimization and the MIST method on three different datasets.

Dataset	Modality	Data (# tiles)	Stitching time			RMSE		
			FRMIS with Optimization		MIST [9]	FRMIS with Optimization		MIST [9]
			MST	SPT		MST	SPT	
**Tak**	Bright-field	026-01-91 (100)	**60.95**	**60.95**	139.49	5.04e-2	4.89 e-2	**4.84e-2**
		051-04-80 (100)	**61.17**	**61.17**	132.57	6.07e-2	5.99e-2	**5.90e-2**
		156-01-86 (100)	**59.73**	**59.73**	132.08	6.80e-2	6.74e-2	6.59e-2
		194-01-70 (100)	**60.05**	**60.05**	135.62	7.09e-2	7.12e-2	**7.01e-2**
		234-01-67 (100)	**55.53**	**55.53**	134.95	8.51e-2	**8.47e-2**	8.49e-2
		31-01 (100)	**58.31**	**58.31**	136.98	**3.87e-2**	3.88e-2	4.03e-2
		33-03 (100)	**57.77**	**57.77**	137.18	**6.17e-2**	6.21e-2	6.27e-2
		36-01 (100)	**57.20**	**57.20**	137.80	6.06e-2	**6.03e-2**	6.22e-2
		49-01 (100)	**58.72**	**58.72**	136.04	5.26e-2	**5.20e-2**	5.50e-2
		53-03 (100)	**58.30**	**58.30**	137.31	**6.59e-2**	**6.59e-2**	7.17e-2
**Stem cell colony**	Phase-contrast	level1 (100)	**15.05**	**15.05**	40.21	2.8e-3	2.7e-3	**2.6e-3**
		Phase (100)	**15.27**	**15.27**	43.64	1.8e-3	1.8e-3	**1.7e-3**
		Small_phase (25)	**4.16**	**4.16**	11.64	2.0e-3	1.8e-3	**1.6e-3**
	Fluorescence	level3 (552)	**98.38**	**98.38**	261.13	6.81e-4	6.83e-4	**6.75e-4**
		level2 (100)	**17.50**	**17.50**	45.79	5.34e-4	5.37e-4	**5.08e-4**
		Small_fluorescent (25)	**4.94**	**4.94**	11.08	**8.31e-4**	8.48e-4	8.32e-4
**Human colon**	Fluorescence	Human colon (609)	**73.77**	73.78	214.81	**6.92e-2**	6.96e-2	NaN
